# Nano-assembly of amyloid β peptide: role of the hairpin fold

**DOI:** 10.1038/s41598-017-02454-0

**Published:** 2017-05-24

**Authors:** Sibaprasad Maity, Mohtadin Hashemi, Yuri L. Lyubchenko

**Affiliations:** Department of Pharmaceutical Sciences, University of Nebraska Medical Center, Omaha, Nebraska United States

## Abstract

Structural investigations have revealed that β hairpin structures are common features in amyloid fibrils, suggesting that these motifs play an important role in amyloid assembly. To test this hypothesis, we characterized the effect of the hairpin fold on the aggregation process using a model β hairpin structure, consisting of two Aβ(14–23) monomers connected by a turn forming YNGK peptide. AFM studies of the assembled aggregates revealed that the hairpin forms spherical structures whereas linear Aβ(14–23) monomers form fibrils. Additionally, an equimolar mixture of the monomer and the hairpin assembles into non-fibrillar aggregates, demonstrating that the hairpin fold dramatically changes the morphology of assembled amyloid aggregates. To understand the molecular mechanism underlying the role of the hairpin fold on amyloid assembly, we performed single-molecule probing experiments to measure interactions between hairpin and monomer and two hairpin complexes. The studies reveal that the stability of hairpin-monomer complexes is much higher than hairpin-hairpin complexes. Molecular dynamics simulations revealed a novel intercalated complex for the hairpin and monomer and Monte Carlo modeling further demonstrated that such nano-assemblies have elevated stability compared with stability of the dimer formed by Aβ(14–23) hairpin. The role of such folding on the amyloid assembly is also discussed.

## Introduction

The self-assembly of amyloid proteins into nano-aggregates is currently considered the main molecular mechanism leading to the development of early onset Alzheimer’s disease and other amyloid-type neurodegenerative diseases^1–3^. The aggregation process is accompanied by a change in the secondary structure of the monomers, eventually leading to the assembly of the fibrillar structures found in amyloid plaques^4–6^. Solid-state NMR studies of amyloid fibrils revealed that cross β structures with either a parallel or an antiparallel arrangement of monomers are the common structural features of fibrils^7, 8^. Another feature of fibrils is the presence of ‘β hairpin’ motifs^9, 10^, such as the turn-like structure from residues 26 to 30 in Aβ42 fibrils^11^. Recently, Maiti *et al*. have identified β hairpin structures in Aβ40 oligomers using surface enhanced Raman spectroscopy and solid-state NMR studies^12^. Similarly, a turn like conformation has been found in Aβ42 oligomers within residues 25–29^13^. Recently, the structure of a β-hairpin conformer of an Aβ40 monomer was stabilized by an antibody, suggesting that the hairpin structure could be an intermediate during Aβ aggregation^14^. It has also been proposed that the turn conformation in Aβ is an early folding event during Aβ fibril nucleation^15, 16^. Together, these findings suggest that the β-hairpin structure is a common motif in amyloid aggregates, however the role of the hairpin conformation in the assembly of oligomers and aggregates remain elusive. Among aggregates, the oligomeric rather than fibrillar forms are considered as the more neurotoxic species^13^. Hence, studying the structure of these aggregates and elucidating the mechanism of how the self-assembly process occurs is crucial for the development of appropriate therapeutic and diagnostic tools for amyloid diseases.

Recent studies have demonstrated that single-molecule approaches are an effective method to study oligomers^17–20^. Previously, we developed an AFM force spectroscopy approach that allowed us to probe the assembly of amyloid proteins and peptides in dimers^21–23^; a major finding of which being that amyloid dimers are highly stable. These findings were further supported by directly measuring the lifetime of dimers by using a single-molecule fluorescence approach termed the tethered approach for probing of intermolecular interactions (TAPIN)^24, 25^. Computational modeling approaches such as molecular dynamics (MD) simulations were also used during structural studies of amyloid oligomers^26, 27^. We applied MD simulation to characterize Aβ(14–23) assembled into dimers and developed a computational approach based on the Monte Carlo simulations enabling us to characterize the structure of Aβ(14–23) dimers probed by the AFM pulling experiments^28^.

In the current study, we address the question of how the hairpin type secondary structure of amyloid β contributes to the amyloid assembly process. A β hairpin amyloid structure was constructed by connecting two Aβ(14–23) monomers with a turn forming YNGK tetra peptide. The experimental studies reveal that the hairpin fold plays a dramatic role in the self-assembly process of Aβ(14–23) peptides. The Aβ(14–23) hairpin formed more stable complexes compared to those formed by Aβ(14–23) monomers. Computational modeling of the Aβ(14–23) hairpin and monomer complexes reveals a sandwich type structure in which the monomer intercalates into the hairpin, which is accompanied by an increased stability. The role of such transiently formed hairpin folds on the aggregation process of amyloid proteins is discussed.

## Results

### Morphological analysis of aggregates

**Figure 1:**
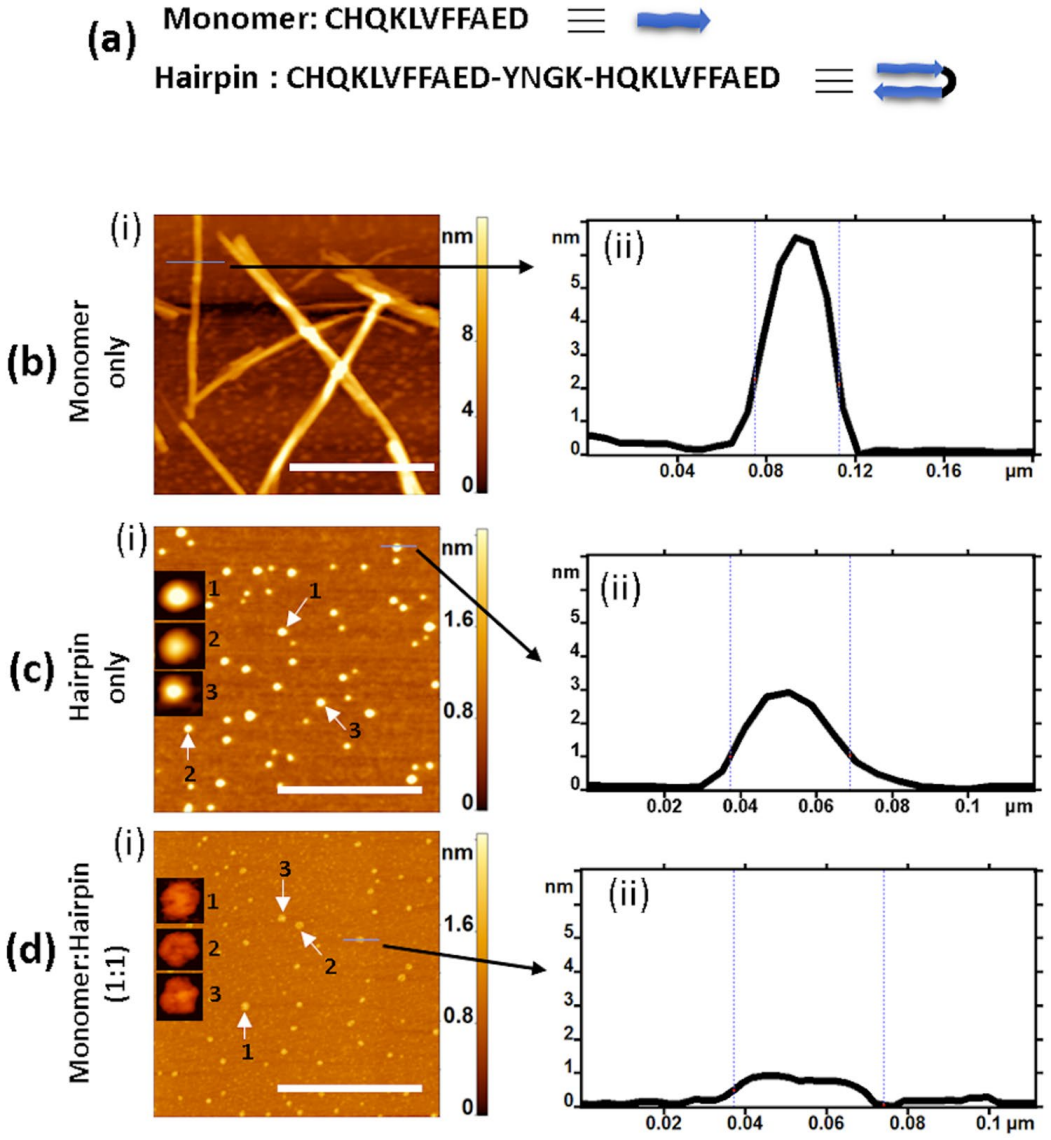
Aggregation study; (**a**) Sequence of Aβ(14–23) monomer and hairpin. **(b**, i), (**c**, i) and (**d**, i) show AFM images of the aggregates from the monomer, the hairpin and the 1:1 mixture of monomer-hairpin, respectively. The insets in (**c**) and (**d**) are zoomed images of the features highlighted by white arrows. The cross section diagrams are shown in (**b**, ii), (**c**, ii) and (**d**, ii) for the selected features (indicated by black arrow) in (**b**, i), (**c**, i) and (**d**, i), respectively. The scale bars are 500 nm.

The sequence of Aβ(14–23) peptide, known to play a critical role in the aggregation of full size Aβ amyloid proteins, is shown in Fig. 1a (top). In addition to this peptide, which is referred to here as a monomer (M), we constructed a tail-to-head configuration by connecting two monomer units with the tetra peptide YNGK. NMR studies^29^ have previously shown that this motif adopts a U-turn geometry, which facilitates the assembly of two monomers in an antiparallel orientation, referred to here as a hairpin (H; Fig. 1a, bottom). As detailed above, we hypothesize that this hairpin structure plays a critical role in the self-assembly/aggregation processes.

To test our hypothesis, we performed aggregation experiments with Aβ(14–23) monomers (100 μM), hairpins (100 μM), and their equimolar mixture (100 μM each), and then imaged the aggregates with AFM. The AFM images for the monomer, hairpin, and the 1:1 mixture of monomer and hairpin are shown in Fig. 1b,c and d, respectively. The monomers form long fibrils (Fig. 1b(i)) with a diameter of 46 nm and a height of 6 nm, as determined from the fibril cross section (highlighted with an arrow; Fig. 1b(ii)), which is consistent with our recent observations^28^. The AFM topographic image in Fig. 1c(i) and the cross section diagram in Fig. 1c(ii) reveal that the hairpin species form globular aggregates. Statistical analysis revealed that height of these globular nanostructures is 2.65 ± 0.61 nm (see Supplementary Fig. S1a). The equimolar mixture of monomer and hairpin also assemble into aggregates with a globular shape, however, their morphology is different from those formed by the hairpin alone (Fig. 1d(i)). The zoomed insets in Fig. 1d and the cross section (Fig. 1d(ii)) evince that the aggregates are disk-shaped. Statistical analysis reveals that the disk-shaped aggregates have a height of 0.85 ± 0.08 nm (see Supplementary Fig. S1b), which is much lower than hairpin aggregates. Note that no fibrillar aggregates were observed in the H-M sample despite the presence of monomer in the reaction mixture.

These aggregation experiments (Fig. 1) demonstrate that the morphology of amyloid assemblies is sensitive to the structure of the monomeric precursors. While native monomers assemble into fibrils (Fig. 1b) like those observed for full size Aβ proteins, the presence of non-native species can produce vastly different morphologies. The hairpin peptide forms globular features, like those for GNNQQNY peptide^30^, with no evidence of the formation of fibrils or protofibrils (Fig. 1c). Similar results were also obtained from the Aβ42 hairpin, which was stabilized by formation of a disulfide bond formed between mutations at A21C and A30C^31^. To further elucidate the role of these hairpin structures on the assembly processes, a set of single-molecule studies along with computational analyses were performed.

### Stability of complexes probed with TAPIN

**Figure 2:**
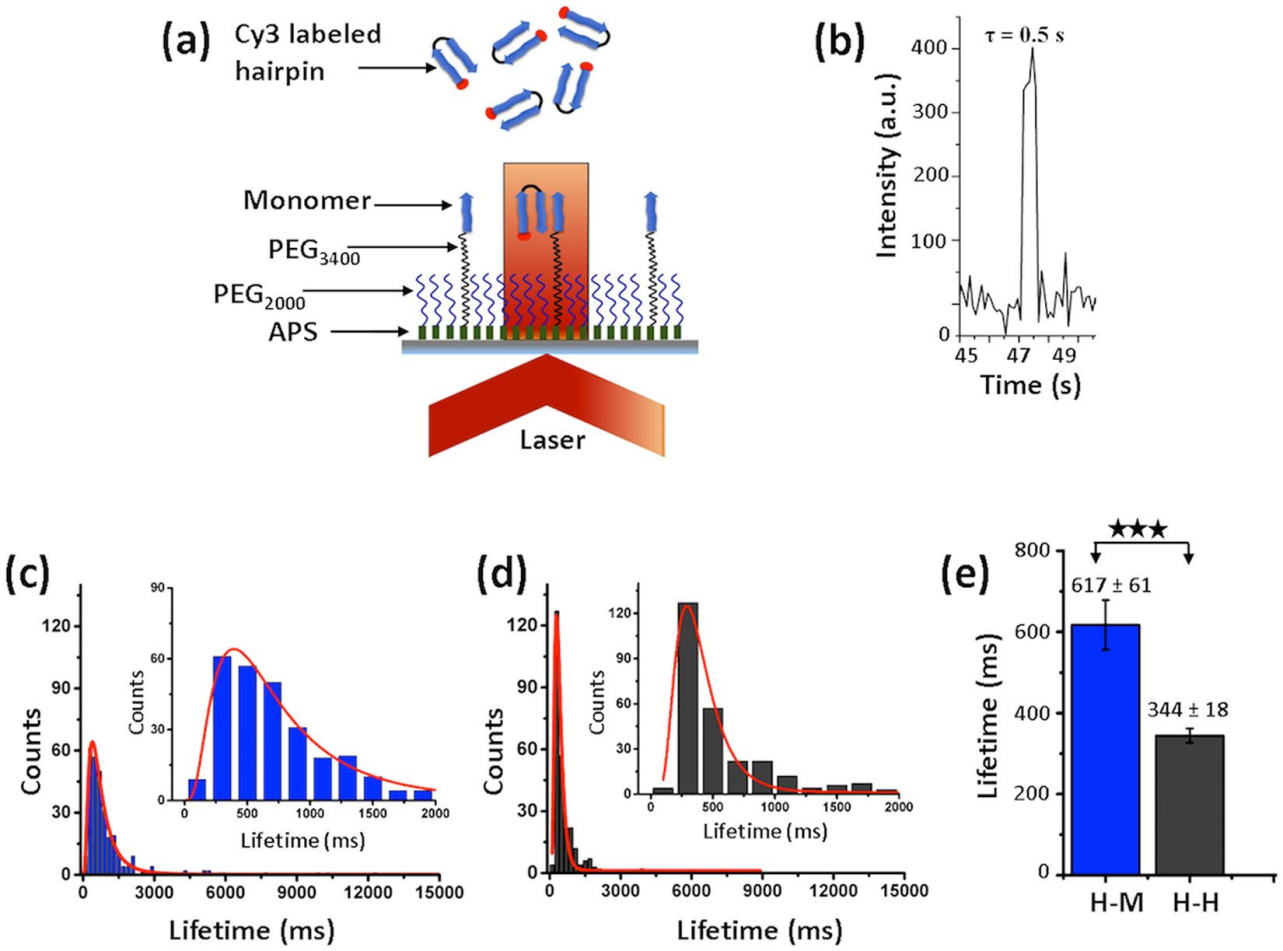
Lifetime measurements for H-M and H-H complexes; (**a**) Schematic representation of TAPIN experimental set up. (**b**) A typical fluorescence time trajectory. (**c**) and (**d**) indicate typical lifetime histograms for H-M (n = 297, blue) and H-H complexes (n = 274, dark grey), respectively. The insets show zoomed histograms in the range of 0–2000 ms. The histograms were fitted with the Lognormal function (red line). (**e**) The average lifetime for the H-M and H-H complexes, extracted from three independent experiments at pH 7.0 and room temperature (mean ± S.D.); the asterisks indicate statistical significance between the two groups using the Kolmogorov-Smirnov test (***p < 0.005). n is the number of data points.

The stability of dimers formed by monomers and hairpins was characterized using TAPIN methodology^25^. This approach utilizes single-molecule florescence in total internal reflection fluorescence (TIRF) mode, allowing us to measure the lifetime of hairpin-monomer (H-M) and hairpin-hairpin (H-H) complexes. The setup is illustrated schematically in Fig. 2a, which depicts the probing of H-M complexes. The H-M complex is formed between a monomer immobilized on the cover slip and a fluorescently labeled hairpin (H) in solution. Probing of the H-H complex is accomplished by immobilizing the hairpin to the cover slip. The molecules were immobilized on the glass coverslip via a flexible PEG tether to allow the tethered peptides to adopt any orientation during complex formation. The assembly and dis-assembly events during complexation can be seen in Movie S1 and S2 (see Supporting information online). Complex formation leads to the appearance of a fluorescence burst and the duration of the signal (a pulse) is used to measure the lifetime of the complex. Figure 2b shows a single fluorescence time trajectory, which corresponds to a complex with a lifetime of 0.5 seconds.

Hundreds of one-step events were analyzed from each set of experiments and assembled as histograms in Fig. 2c and d for the H-M and H-H complexes, respectively. The insets in these figures indicate zoomed histograms in the time range of 0–2000 millisecond (ms), illustrating that most of the observed events are within this lifetime range. The average lifetimes of H-M and H-H complexes were obtained after approximation of the histograms with the Lognormal function^32^. The calculated average lifetime for a H-M complex is 617 ± 61 ms (mean ± standard deviation), while the average lifetime for a H-H complex is 344 ± 18 ms (mean ± standard deviation) (Fig. 2e, Table 1); thereby revealing that H-M complexes are two times more stable than H-H complexes. Reproducibility of lifetime measurements was demonstrated by performing three independent experiments for each system in similar experimental conditions (see Supplementary Fig. S6, Table S1). A number of optimization and control experiments e.g. photo physical properties of the dye and non-specific adsorption of fluorophore labeled hairpin to the working surface, were also performed (see Supplementary Figs S3–5, Movie S3).

**Table 1:** Average lifetime and rupture force values from three different single-molecule methods at neutral pH (7.0). Values are represented as mean ± S.D.

Probing systems	Lifetime (ms)	Rupture force (pN)	Computational MCP force (pN)
H-M complex	617 ± 61	164 ± 17	141 ± 12
H-H complex	344 ± 18	100 ± 6	75 ± 10

### Strength of complexes measured by AFM force spectroscopy

**Figure 3:**
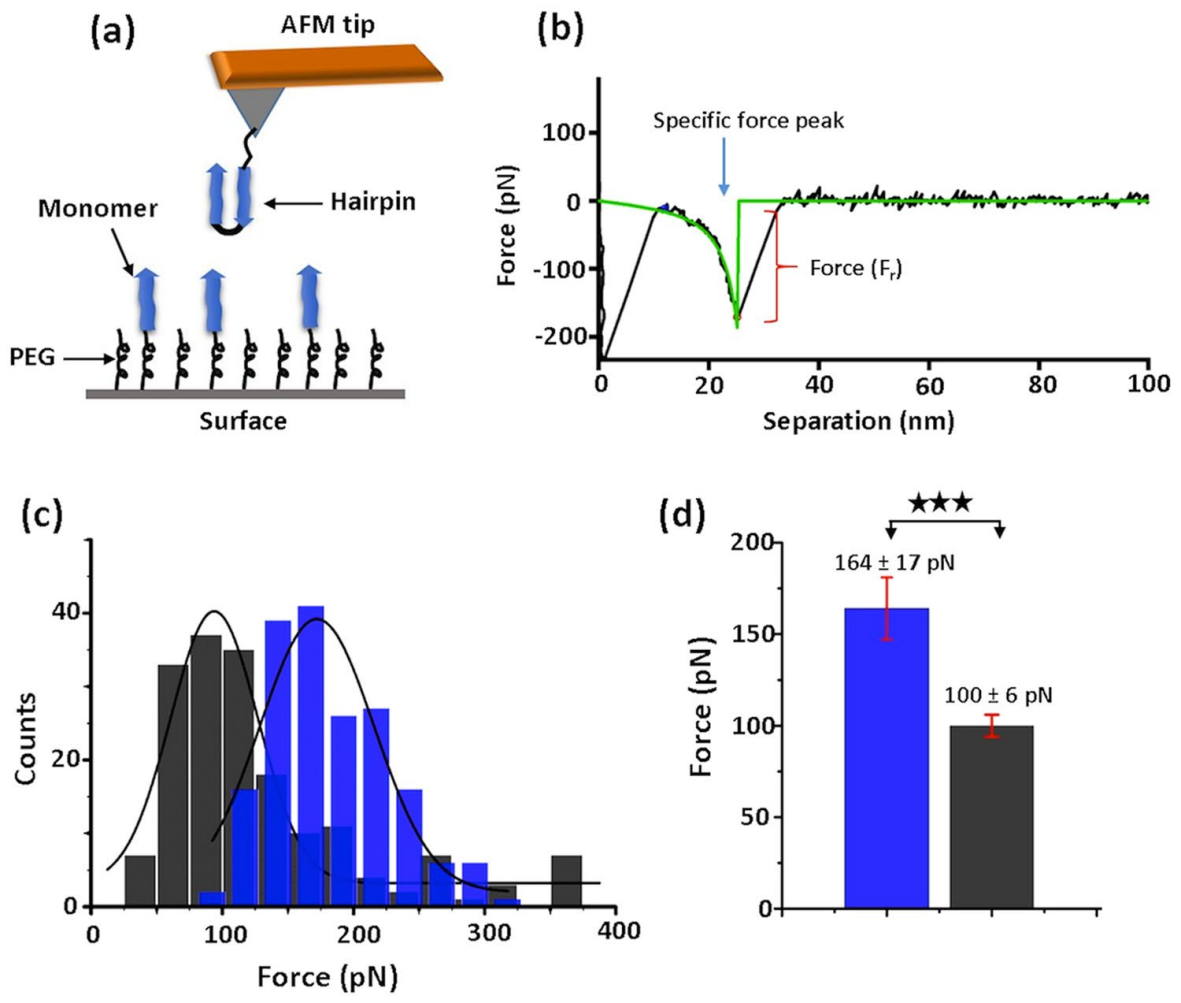
AFM force measurement; (**a**) Schematic of force spectroscopy setup for H-M assembly. (**b**) A typical force-distance (F-D) curve; the specific unbinding fingerprint is highlighted with an arrow. The green line indicates worm-like-chain (WLC) fitting that estimate the rupture force (Fr). (**c**) Force histograms for H-M (n = 180, blue) and H-H (n = 175, dark grey) complexes; black lines indicate Gaussian fitting. (**d**) Bar diagram showing the mean rupture force for H-M (blue) and H-H (grey) assemblies from three independent experiments. Asterisks indicate statistical significance between the two groups using Kolmogorov-Smirnov test (***p < 0.005). The experimental conditions were as follows: retraction speed, 500 nm/s; buffer, 10 mM sodium phosphate buffer (pH 7.0); room temperature. n is the number of data points.

The strength of H-M and H-H complexes was characterized using an AFM based single-molecule force spectroscopy approach. Figure 3a illustrates the experimental setup for AFM force measurements and depicts the H-M assembly. The hairpin was covalently attached to the AFM tip via a flexible PEG tether. Probing of the H-M complex was performed by approaching the hairpin functionalized AFM tip to the monomer functionalized mica surface; the assembled complex was then pulled apart by retracting the tip. Similarly, H-H complexes were probed by multiple approach-retraction steps of the hairpin-functionalized tip to the hairpin-functionalized surface. Figure 3b shows a typical force-distance (F-D) curve that has a specific intermolecular interaction peak (indicated with arrow).

Typically, several thousand approach-retraction cycles were performed for each set of experiments, and force curves with the complex rupture fingerprint (~8–10% of total approach-retraction cycles) were selected. The reproducibility of the events was confirmed by overlay of F-D curves, as shown in Fig. S7 (see Supporting information online). Each force curve was approximated with the worm-like-chain model^33^ (WLC, equation 1). The force required to dissociate the complexes (rupture force, F_r_) and the distance to the rupture events, as measured by the contour length, L_c_, of the tethers, were estimated from this fitting^23, 34^. Several hundred specific rupture events were analyzed and the values of the rupture forces and contour lengths were assembled as histograms.

Contour length analysis is shown in Fig. S8 (see Supporting information online). The histograms, approximated with Gaussians and averaged over data from three independent experiments, produced values of 31 ± 1 nm and 31 ± 4 nm for the H-M and H-H complexes, respectively. These numbers agree with the expected L_c_ value of ~ 33 ± 5 nm (the error indicates the PEG tether length polydispersity).

Histograms of rupture force values for H-M and H-H complexes drawn in blue and grey colors, respectively, are shown as Fig. 3c. The average force was estimated by fitting the histograms with the Gaussian distribution function, depicted as a black line. The experiments were repeated three times and the results of the force distributions for both complexes are presented in Supplementary Fig. S9. The mean values of the rupture forces are assembled in Fig. 3d and Table S2 (see Supporting information online) to demonstrate the reproducibility of the data. Force spectroscopy experiments showed that the value for the rupture force for H-M complexes is 164 ± 17 pN (mean ± standard deviation), which is higher than the force value for H-H complexes, 100 ± 6 pN (mean ± standard deviation). Therefore, the force spectroscopy data demonstrate that H-M complex strength is higher than that of H-H.

### Structure and dynamics of dimers revealed by molecular dynamics simulations

The structures of the H-M and H-H complexes were further investigated by computational modeling. H-M complexes were studied by simulating the interaction of a monomer with the hairpin for 2.4 μs using conventional MD (cMD) simulations, followed by 500 ns of accelerated MD (aMD) simulation in order to extend the conformational sampling efficiency by several orders of magnitude^35^.

**Figure 4:**
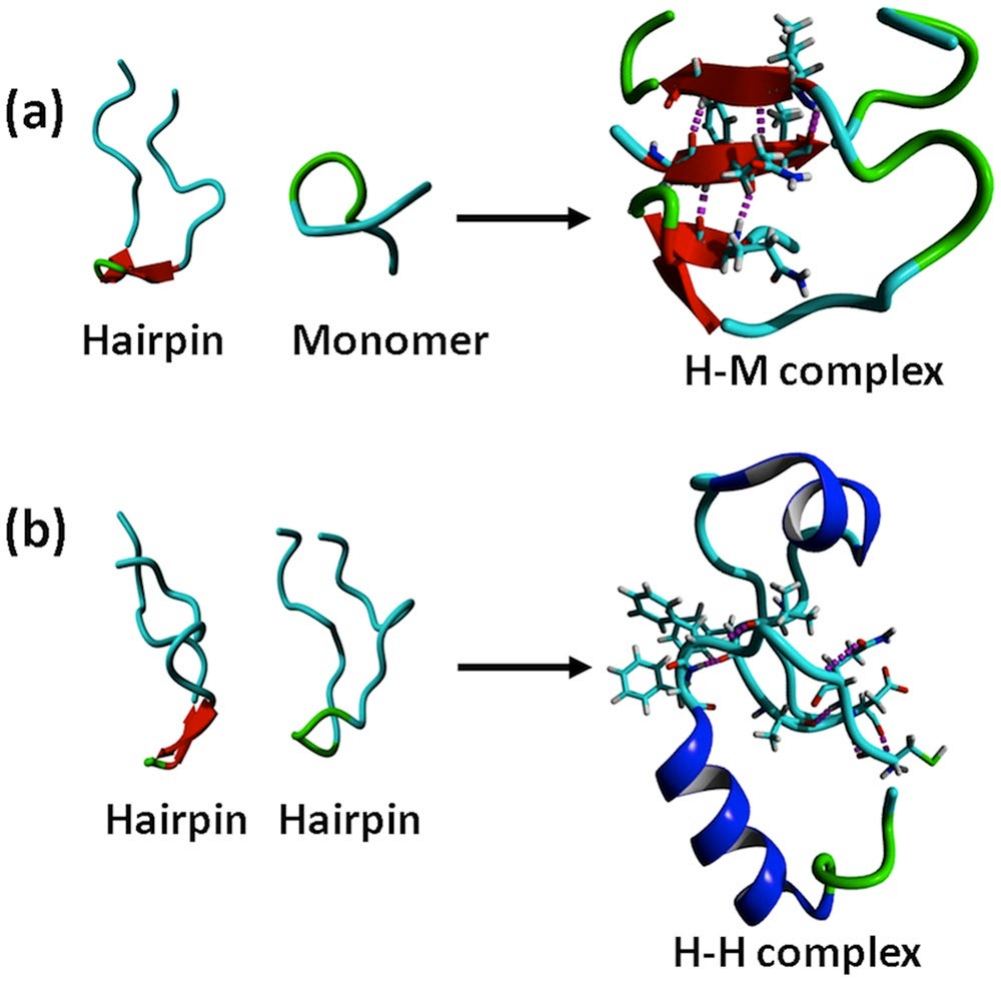
Structural investigation by molecular dynamics simulation; (**a**) Initial structure of H-M complex (left) and the most likely conformation for the H-M complex (right). (**b**) H-H complexes; initial structure (left) and the predicted most likely structure (right).

Analysis of the free energy landscape plot (FEP), based on the dihedral principal component analysis (dPCA)^36^, reveals several minima, indicating the conformational heterogeneity of H-M complexes (see Supplementary Fig. S12). Further investigation of the conformations in these energy minima by performing Monte Carlo pulling (MCP)^37^ simulations revealed the most likely structure of the H-M complex, presented in Fig. 4a. The identified structure is a novel conformation with the monomer intercalated inside the hairpin. This intercalated structure is formed from four short β-strands, resulting in an extended antiparallel arrangement, which is stabilized by three intra-molecular and five inter-molecular hydrogen bonds. Moreover, side chain interactions between Phe and His residues and a Lys-Asp salt bridge also contribute to the stability of the complex. These interactions contribute to the elevated stability of the intercalated H-M complex as seen in MCP experiments, which revealed a mean rupture force of 141 ± 12 pN (see Supplementary Fig. S16c). Hence, we posit that the interlaced structure is the main structural motif of the H-M complex, since the MCP rupture force is close to experimental value (141 ± 12 pN vs. 164 ± 17 pN).

Similar strategy was employed to characterize the H-H complex. The most probable conformation of H-H dimer is presented in Fig. 4b. This structure, when probed by MCP simulations, dissociates at a mean force of 75 ± 10 pN (see Supplementary Fig. S16c). These data show that H-H complex is stabilized by four intermolecular hydrogen bonds and by side chains from both hairpins interacting to form a hydrophobic pocket in the complex consisting of LVFFA residues from both peptides. Hence computational modeling suggests that H-M and H-H complexes assemble very differently. The H-H dimers interact side-by-side fashion, whereas H-M dimers are formed by intercalation of the monomer into the hairpin. Importantly, H-M assembly is considerably more stable compared with the side-by-side H-H complex.

## Discussion

The data presented in this multifaceted study provides compelling evidence to support our hypothesis, that the folding pattern of amyloid protein defines the aggregation pathway. Aggregation studies demonstrate that the hairpin construct of Aβ(14–23) peptide forms spherical structures in contrast to the fibrils formed from monomers. Importantly, the equimolar mixture of hairpins and monomers does not produce fibrils; rather these species assemble into disk-shaped nanostructures. The finding that fibrillar aggregates do not form suggests that the hairpin fold dramatically changes the aggregation pattern despite the presence of the fibril forming monomers. An explanation for this behavior is well justified by the single-molecule experiments.

First, AFM force spectroscopy measurements (Fig. 3) demonstrate that the strength of hairpin-monomer interactions is considerably higher than that of hairpin-hairpin interactions. The data is fully in line with the lifetime measurements which show that hairpin-monomer assembly has a longer lifetime compared to the hairpin-hairpin assembly (Fig. 2). By applying an all-atom molecular dynamics simulation to the system, we structurally characterized the interactions that resulted in the observed lifetimes of the nano-assemblies. The extended MD simulations revealed a novel intercalated type complex for the hairpin-monomer (Fig. 4). Monte Carlo based simulation of the AFM pulling experiments further demonstrated that the intercalated assembly produces a high rupture force value that is in good correlation with the experimental AFM probing of the hairpin-monomer interactions. Altogether, experimental data along with computational analyses reveals that the secondary structure of the hairpin provides a novel type of interaction with the monomer. This novel assembly explains the results of the aggregation experiments in which an equimolar mixture of monomers and hairpins produced the intriguing observation of non-fibril assemblies. In the mixture of monomers and hairpins all the combinations of H-H, H-M and M-M are possible, but experimental data and computational modeling suggest that the H-M arrangement produces the most stable dimer. As a result, the most stable H-M configuration in the mixture acts to seed the aggregation process.

The observation that the morphology of aggregates is highly dependent on the secondary structure of Aβ(14–23) peptide suggests that the conformational transition of the full-size Aβ peptide during the aggregation process plays a crucial role in the entire aggregation process of amyloids. Given the fact that Aβ(14–23) forms fibrils with morphologies similar to those for full size Aβ protein, we assume that this segment in the full size Aβ42 monomer should be structured to allow the molecules to assemble oligomers capable of fibril formation. Alternatively, intramolecular folding of the 14–36 segment of Aβ42 protein in the hairpin-type structure can lead to the assembly of non-fibrillar aggregates^38, 39^. These can be morphologically similar to those found in Fig. 1c and d, due to the structural heterogeneity of the monomers containing a mixture of folded and unfolded molecules, respectively. However, these are hypothetical models that need to be verified through future investigation. The design of appropriate experimental and computational studies and the use of the approaches described in this paper will be of value in these future studies.

Increasing evidence suggests that the self-assembly of Aβ protein underlies the early onset of AD. Given that small Aβ nano-assemblies (oligomers) are the most neurotoxic species, a shift to an aggregation pattern dominated by assembly of non-fibrillar species would shift the aggregation process to the disease prone state. Based on our studies, we hypothesize that stabilization of the internal hairpin structure within (14–36) segment of the full size of Aβ42 protein can drive such a process. Furthermore, this assembly can be modulated by environmental conditions or interaction of Aβ42 protein with other molecules including cellular membranes. Our experimental approaches can be used for testing this hypothesis.

Overall, our study provides new insights into the role of the monomer structure of amyloid peptides on the self-assembly process that contributes to the formation of disease related aggregates. Importantly, the developed experimental approaches and validation approaches for computational analyses are not limited to amyloid proteins, but can also be applied to other molecular systems.

## Materials and Methods

### Materials

Peptides [CHQKLVFFAED and CHQKLVFFAED-YNGK-HQKLVFFAED] were purchased from Peptide 2.0 Inc. (VA, USA). N-hydroxysuccinimide (NHS) and maleimide (MAL) functionalized Cyanine3 dyes (Cyanine3-NHS and Cyanine3-MAL) were purchased from Lumiprobe Corporation (Florida, USA). MAL-PEG-SVA (M.W 3400 g/mol) and mPEG-SVA (M.W 2000 g/mol) were from Laysan Bio (Arab, AL), Tris-(2-Carboxyethyl) phosphine, Hydrochloride (TCEP) was from Hampton Research Inc. (CA, USA), β-mercaptoethanol and 1,1,1,3,3,3 Hexafluoroisopropanol (HFIP) were purchased from Sigma-Aldrich, USA. The synthesis of maleimide-polyethylene glycol-silatrane (MAS) and 1-(3-aminopropyl) silatrane (APS) was described in our previous publication^40^.

### Preparation of peptide stock solution

A measured amount of peptides were dissolved and sonicated for 5 min in 100 μL of 1,1,1,3,3,3 Hexafluoroisopropanol (HFIP) to destroy pre-aggregated oligomers^41^. The solvent was then evaporated in a vacuum for 4 hours. Stock solutions were prepared in DMSO (concentration 2 μM) and stored at −20 °C until use. The stock solutions were used within 7 days to avoid aggregation.

### Aggregation study

Each of the monomer and hairpin was dissolved in HFIP and sonicated for 5 minutes to destroy pre-aggregated oligomers. The solvent was then evaporated completely and then dissolved in 10 mM sodium phosphate buffer (pH 7.0) to obtain 100 μM solution. Three samples were prepared (1) 100 μM monomer (2) 100 μM hairpin and (3) 1:1 mixture of 100 μM monomer-hairpin mixture. The samples were incubated at room temperature for 3 days, after which aliquots were taken out from the test tubes and prepared for AFM imaging. The specimens were imaged with Multimode III in tapping mode using TESPA AFM probes (nominal spring constant 42 N/m; AFM and the probes both from Bruker Corporation, USA). The images were processed with Femtoscan Online AFM software (Advanced Technologies Center, Lomonosov Moscow State University, Moscow).

### Sample preparation for TAPIN experiment

The sample preparation steps were as described in ref. 24. Typically, glass coverslips were cleaned with chromic acid for 30 min, followed by multiple rinses with water. The coverslips were then assembled into a sample holder (PicoQuant, Berlin, Germany), by placing the coverslip at the bottom, then a 0.1-mm-thick teflon spacer, and finally a 25-mm-diameter quartz disk that contains two pin holes for injecting liquid samples. The chamber was filled with 167 mM APS solution for 30 min, followed by multiple rinses with water. The chamber was then filled with a mixture of MAL-PEG-SVA (MW 3400 g/mol)/mPEG-SVA (MW 2000 g/mol) with a molar ratio of 1:60 in 10 mM bicarbonate buffer, pH 8.0 for 1 h (see Supplementary Fig. S3). 50 pM of Aβ(14–23) monomer or hairpin was treated with TCEP to destroy S-S bonds (if any), followed by rinsing with water. Two samples were prepared each time. Before adding the fluorescently labeled peptides to the chamber, the surfaces were exposed to working laser (wavelength 532 nm) for 20 min to bleach any fluorescently active contamination. The hairpin was labeled with fluorophore (Cy3) by reacting Cy3-maleimide with the hairpin. The details of synthesis and purification are discussed in the supporting information (see Supplementary Fig. S2).

### Lifetime data acquisition and analysis

Single-molecule fluorescence imaging was performed with an objective-type TIRF microscope built around an Olympus IX71 microscope (Hitschfel Instruments, St. Louis, MO). A detailed description of the system is provided in ref. 42. An oil-immersion UPlanSApo 100x objective with 1.40 NA (Olympus, Tokyo, Japan) was used for all measurements. A laser line at 532 nm (ThorLabs Inc., New Jersey, USA) was used to excite Cy3 labeled peptides in TIRF mode. The laser intensity was set to 260 mA for all experiments. Fluorescence emission was detected with an electron-multiplying charge-coupled-device camera (ImageEM Enhanced C9100–13, Hamamatsu, Bridgewater, NJ). TIRF videos of 2–3 min were recorded at a temporal resolution of 100 ms.

TIRF images were processed and analyzed with Slidebook 5.0 software (Intelligent Imaging Innovations (3i), Denver, CO). Whenever a complex was detected, a confined area was circled out for further analysis. Lifetimes were analyzed from fluorescence time trajectories. A typical complex showed a sudden increase of fluorescence intensity, with an abrupt drop back to background after a short dwell time. Several hundred events were analyzed for each experiment and data were assembled into histograms. The average lifetimes were estimated by fitting the histograms with Lognormal function. To ensure reproducibility and enhance the accuracy of the measured lifetimes, each H-M and H-H probing experiment was repeated in triplicate. The average lifetime was calculated as mean ± standard deviation.

### AFM tip and mica surface functionalization

The peptide immobilization procedures on the AFM tip and mica surfaces were performed as described in ref. 30. Briefly, AFM probes (MSNL, Bruker, CA) were cleaned with ethanol and water, followed by treatment with UV (λ_366_ nm) light for 45 min. The probes were dipped into 100 μM of maleimide-polyethylene glycol-silatrane (MAS) for 1 h and rinsed with water to cover the tip with maleimide functional groups. The stock peptide solutions were diluted to 20 nM in 1 μM TCEP solution (to destroy any S-S bonds between the peptide) in 10 mM sodium phosphate buffer (pH 7.0). The probes were incubated in this solution for 1 h, followed by rinsing with water. Finally, the unreacted maleimide groups were quenched with 10 mM β-mercaptoethanol, rinsed, and stored in water at 4 °C until needed. Typically, the storage time was less than 24 h.

The freshly cleaved mica surface, glued to the glass slide, was treated with 167 μM APS solution for 30 min, and then with 167 μM MAL-PEG_3400_-SVA in DMSO for 1 h, followed by multiple rinses with DMSO and water. Next, the surfaces were covered with 40 nM of hairpin solution (pretreated with TCEP) for 2 h. Finally, unreacted maleimide groups were quenched with β-mercaptoethanol, rinsed, and stored at 4 °C until needed. Typically, the storage time was less than 24 h.

### Force measurements and data analysis

All AFM force experiments were done on the Asylum MFP-3D (Oxford Instruments, Santa Barbara, CA) using silicon nitride cantilevers (MSNL, Bruker, CA) with the spring constant in the range of 20−30 pN/nm, measured by thermal method. All the measurements were performed at room temperature in 10 mM sodium phosphate buffer (pH 7.0) with a retraction speed of 500 nm/s. For each pulling experiment, several thousand force-distance (F-D) curves were collected at different locations on the mica surface (5000 nm × 5000 nm grid). Out of this set, force curves corresponding to the characteristic rupture events were selected (a typical yield was about 8–10%). The selected force curves were approximated with the worm-like-chain (WLC) model (Equation 1) that estimate rupture force (Fr) and contour length (Lc), as shown below.

1 $$F(x)=\frac{{k}_{B}T}{{L}_{p}}[\frac{1}{4}{(1-\frac{x}{{L}_{c}})}^{-2}-\frac{1}{4}+\frac{x}{{L}_{c}}]$$

where F(x) is the force at the distance of x, k_B_ is the Boltzmann constant, T is absolute temperature, and Lp and Lc are the persistence length and contour length, respectively.

The data were assembled into histograms and fitted with the Gaussian function to estimate the most probable force and contour length for the specific rupture events. Control experiments, during which the AFM tips were functionalized with peptides but the surface only had PEG (no peptide), were performed. In these control experiments, only 0.2–0.4% of the F-D curves showed peaks at a short range, but those were not similar to the nonlinear PEG extension. Additionally, the measured force values were in the range of the instrument noise (10–20 pN), which further confirmed the specificity of our experimental setup.

### Computational modeling

To generate the initial structure of the hairpin that was used for the H-M and H-H complex simulations, we conducted MD simulations using the Amber ff99SB-ILDN force field^43^ and the TIP3P water model^44^. The initial hairpin structure was created by placing the amino acids in a linear and fully stretched conformation. To mimic the experimental design, a Cys residue was added to the N-terminus. The structure was then solvated in a cubic box with TIP3P water molecules. The minimum distance between the peptide and the edge of the water box was 1.5 nm, so that any interactions between periodic copies, due to periodic boundary condition, were avoided. The protonation states of Lys and His residues were set to mimic neutral pH conditions. Na^+^ and Cl^−^ ions were added to neutralize the system charge and to keep a constant salt concentration of 150 mM. This conformation was subsequently simulated for 1.2 μs in an NPT (constant Number, constant Pressure, and constant Temperature) ensemble at 1 bar and 300 K. Other details of the simulation setup were adopted from our previous work^28^. This simulation was performed using the special-purpose super computer Anton^45^.

The dihedral Principal Component analysis (dPCA)^36^ was used to acquire the representative structures after the MD simulation. The dihedral angles of the terminal residues were ignored. Equation 2 (below) was used to calculate the free energy:

2 $${\rm{\Delta }}G(V1,V2)=-\,{k}_{B}T\,\mathrm{ln}\,(\frac{P(V1,V2)}{{P}_{max}})$$

where V1 and V2 are the 1st and 2nd largest Principal Components; P(V1, V2) represents the distribution obtained from the MD trajectories, *P*_*max*_ is the maximum value of the distribution; and *k*_*B*_ and *T* are the Boltzmann constant and the absolute temperature, respectively. The Fortran program written by Dr. Yuguang Mu was used to perform this analysis.

The H-M system was assembled using the structure obtained for the Aβ(14–23) hairpin in the previous step and the monomer structure identified in ref. 28. The H-M system was solvated into a cubic box using TIP3P water molecules. In order to allow free tumbling before intermolecular contact, the COM distance of the two molecules was set to 2 nm. All other parameters were the same as the hairpin simulation. The H-H system was assembled in a similar fashion; however, instead of a hairpin and a monomer, the system consisted of two copies of the hairpin randomly placed at a COM distance of 2 nm. Both systems were then simulated for 2.4 μs in an NPT ensemble at 300 K and 1 bar. These simulations were carried out on Crane at the Holland Computing Center (HCC) and Comet at the San Diego Supercomputer Center using the Amber14^46^ software package.

To further extend conformational sampling, the resulting structures from the MD simulations were subjected to the accelerated MD (aMD) simulation method^35^. The two systems were then simulated with a 500 ns aMD simulation as an NVT ensemble at 300 K. The simulations were carried out using Crane and Comet.

The Monte Carlo pulling (MCP) approach with the modified PROFASI package (http://cbbp.thep.lu.se/activities/profasi/) was used to investigate the molecular conformations of the H-M and H-H complexes. Briefly, the two Cα atoms of the N-terminal Cys residues of each molecule were defined as the pulling groups. A virtual spring was attached onto each pulling group and used to pull them along a vector during the pulling process. A detailed description can be found in ref. 47. We mimicked the experimental pulling rate of 500 nm/s, which translates to v = 0.083 for all MCP simulations. The experiments were carried out using Crane and Tusker at HCC.

## Electronic supplementary material

Movie S1:

Movie S2.

Movie S3.

Supplementary info
