# Supplementary material for: Nano-assembly of amyloid β peptide: role of the hairpin fold
Source: Sci Rep. 2017 May 24;7:2344. doi: 10.1038/s41598-017-02454-0 (PMC5443804; doi:10.1038/s41598-017-02454-0)
Supplement: Supplementary file 4 — Supplementary info [file 41598_2017_2454_MOESM4_ESM.pdf]

Supporting Information

Nano-assembly of amyloid β peptide: role of the hairpin fold

Sibaprasad Maity, Mohtadin Hashemi, Yuri L. Lyubchenko*

Department of Pharmaceutical Sciences, University of Nebraska Medical Center, Omaha, Nebraska, United States

Single-molecule fluorescence experiments

Labeling of the Aβ(14-23) monomer and hairpin with Cy3 dye

Cysteine-HQKLVFFAED was weighed to about 0.1 mg with a high precision microbalance (Sartorius AG, Germany), dissolved in 100 μL of 1,1,1,3,3,3 hexafluoro-2-propanol (HFIP) and sonicated for 5 min to break down pre-aggregated oligomers. The solvent was then evaporated in a rotary vacuum (Eppendorf Vacufuge Concentrator, Fischer scientific, USA). The peptide was dissolved in 10 mM sodium carbonate buffer (pH 7.8) and mixed at a molar ratio of 1:1 with freshly prepared Cy3-NHS solution. The solution was then incubated and stirred for 2 h in the dark. The solute was then lyophilized and the product was purified by reverse-phase HPLC (Phenomenex C18 column, 250 mm x 4.6 mm, Solvent A: 0.1% TFA in water, Solvent B: 0.1% TFA in acetonitrile). Control HPLC experiments were also run for the pure peptide and the Cy3-NHS dye with the same solvent gradients to gauge their appropriate product peaks. The pure product was identified

as a new peak in the crude product HPLC spectra, as marked in Fig. S2a. The marked product was collected and confirmed by MALDI-TOF mass spectroscopy (not shown). The percent of conversion was 28% according to HPLC results.

Similarly, cysteine-HQKLVFFAED-YNGK-HQKLVFFAED (hairpin) was labeled with Cy3 by reacting the hairpin with the Cy3-Mal dye. The coupling reaction was performed in 10 mM sodium phosphate buffer pH (7.0) at room temperature for 5 h with a hairpin:Cy3 molar ratio of 1:10. At the end of the reaction, the solute was lyophilized and the product was isolated by reverse-phase HPLC, as described above. The pure product was identified by comparing the HPLC peaks of the crude product with the hairpin peptide and the Cy3-Mal dye, as marked in Fig. S2b. The product was confirmed by MALDI-TOF mass spectroscopy. The percent of conversion was 37% according to the HPLC results.

Control experiments for TAPIN experiments

Optimization of the PEG ratio for surface functionalization during TIRF imaging

Three glass coverslips were cleaned with chromic acid for 30 min and washed thoroughly with water. The surfaces were treated with 167 μM aqueous APS for another 30 min and rinsed with water. The three surfaces were then treated separately with a 167 μM mixture of MAL-PEG-SVA/mPEG-SVA in a molar ratio of either 1:20, 1:60, or 1:100 in 10 mM sodium bicarbonate buffer (pH 8.0) for 1 h and washed with water. A TCEP treated 50 pM Cy3-labeled Aβ(14-23) monomer (with an active thiol group) solution was then deposited on the three surfaces and incubated for 1 h in a dark place to allow the maleimide-thiol reaction to occur. The surfaces were then washed with water and dried under dry argon. TIRF images were recorded from each surface by adjusting the surface on the microscope stage and covering the surface with water. The surface coverage for each sample is shown in Fig. S3. The results indicate that the surface with a 1:20 ratio of the MAL-PEG/mPEG

mixture was too crowded, while the surface with a 1:100 ratio showed only a very few bright spots. However, the surface with a MAL-PEG/mPEG mixture of 1:60 had a reasonable amount of coverage; therefore, this ratio was chosen for further experiments.

Photo-bleaching and blinking tests

Photo-bleaching and blinking experiments were performed to test the photo-physical properties of the Cy3 dye by covalently attaching the Cy3-labeled Aβ(14-23) monomer to the surface and performing TIRF imaging for an extended time. The glass coverslip was cleaned with chromic acid and water, followed by treatment with a 167 µM APS solution. MAL-PEG-SVA/mPEG-SVA mixture (167 µM) with a molar ratio of 1:60 was added to the surface and incubated for 1 h, followed by washing with water. A TCEP treated 50 pM Cy3-labeled Aβ(14-23) monomer solution was added to the surface, which allowed the maleimide-thiol reaction to occur. The surfaces were washed, assembled into the instrument, and 100 µL of 10 mM sodium phosphate buffer was added to the surface. TIRF movies were recorded from the surface for 3 min. The data suggested that no photo-bleaching or blinking occurred during the acquisition (Movie S3). A time trajectory is shown in Fig. S4, which indicates no bleaching or blinking events.

Specific vs. nonspecific binding of the Cy3-labeled hairpin on the modified surface

Specific binding of the fluorophore-labeled hairpin for H-M complex formation was compared with the nonspecific binding of the fluorophore-labeled hairpin with the surface, which does not have any covalently bound monomers. The nonspecific binding of the Cy3-labeled hairpin was tested by placing the fluorophore-labeled hairpin solution on the mPEG-coated surface and recording a TIRF video over time. The glass coverslip was cleaned, treated with APS, and treated with only mPEG-SVA. Note that the coverslip was

not treated with MAL-PEG-SVA. The mPEG-treated surface was washed, placed on the TIRF stage and 1 nM of the Cy3-labeled hairpin solution was added. A TIRF video was then recorded. Only a very few bright spots were observed on the surface, indicative of nonspecific absorption. Fig. S5 indicates a large number of specific interactions on the surface modified with the monomer (Fig. S5a), but only a very few spots were observed on the mPEG-coated surface (Fig. S5b). These results clearly demonstrate that the complexes identified during probing experiments were from specific interactions.

Computational analysis

MD simulation of H-M and H-H complex assembly. We applied all-atom MD simulations with the explicit TIP3P water model to both systems using the approach described in our recent publications[1]. First, we performed MD simulations of the fully stretched hairpin for 1.2 μs to obtain the most probable conformations of the hairpin (Fig. S10 and S11). Next, the most representative hairpin structure was used to assemble the H-M complex with the monomeric structure obtained in reference[1]. In parallel, two copies of the hairpin were used to assemble the H-H complex. Each of the systems were simulated for 2.4 μs using conventional MD (cMD) simulations, followed by 500 ns of accelerated MD (aMD) simulation to extend the conformational sampling efficiency by several orders of magnitude[2]. This simulation scheme was recently used to successfully characterize the interaction of full-size Aβ42[3].

We performed cluster analysis based on the dihedral Principle Component Analysis (dPCA)[4] to determine the structures associated with the H-M complexes (Fig. S12). The monomer is dynamic, undergoing structural transition, where as the hairpin remains in a β sheet conformation for the majority of the simulation (Fig. S13). Fig. S14 shows the results of the simulations for H-H complex formation and its associated dynamics. The

free energy landscape plot (FEP), based on the dPCA method, reveals several minima, indicating the conformational heterogeneity of H-H complexes (Fig. S14). Similar analysis of the H-H complex demonstrating the different conformers, corresponding to the energy minima, and secondary structure transition over time (Fig. S15) clearly indicate that H-H complexes are primarily α-helical.

*Monte Carlo Pulling of H-M and H-H complexes***.** To validate the simulated conformations of the H-M and H-H complexes, we used our previously established validation method called the Monte Carlo Pulling (MCP) approach enabling us to simulate AFM pulling experiments to generate force curves that are compared with the experiment.[5]

To characterize the molecular arrangement in H-M complexes, we used four structures obtained from the dPCA of aMD results that correspond to different minima in the FEP for the H-M system (Fig. S12). We performed 500 MCP experiments for each of the selected structures. The forces generated from each structure were assembled into histograms and compared with experimental data. The MCP results are shown in Fig. S16a. From the MCP simulations, it becomes clear that the intercalated structure (Fig. S16c) forms the most stable complex, with a mean rupture force of 141 ± 12 pN. The distribution of forces and the mean are consistent with experimentally obtained rupture forces, 164 ± 17 pN, (Fig. 3D).

A similar analysis was performed for H-H complexes corresponding to the minima in Fig. S14 and are presented on Fig. S16b. The rupture forces for the H-H complex are almost two times smaller, 75 ± 10 pN, than the rupture forces for the H-M complex. The experimental mean force value is 100 ± 6 pN, which is slightly higher than that obtained in theory. MCP force histogram for the complex matching the experimental results are presented in Fig. S16c.

During rupture, both the H-M and H-H complexes undergo conformational transitions (illustrated in Fig. S17). For the H-M complex, the transitions increase the order of the complex due to re-orientation of the peptide chains that leads to formation of extended β-sheet. The primary stabilizing force of the complex is the backbone and side-chain interactions that stabilize the β-structure. Similar structural re-organization takes place for the H-H complex; as the pulling progresses the complex is re-oriented and forms a compact hydrophobic pocket that stabilizes the complex.

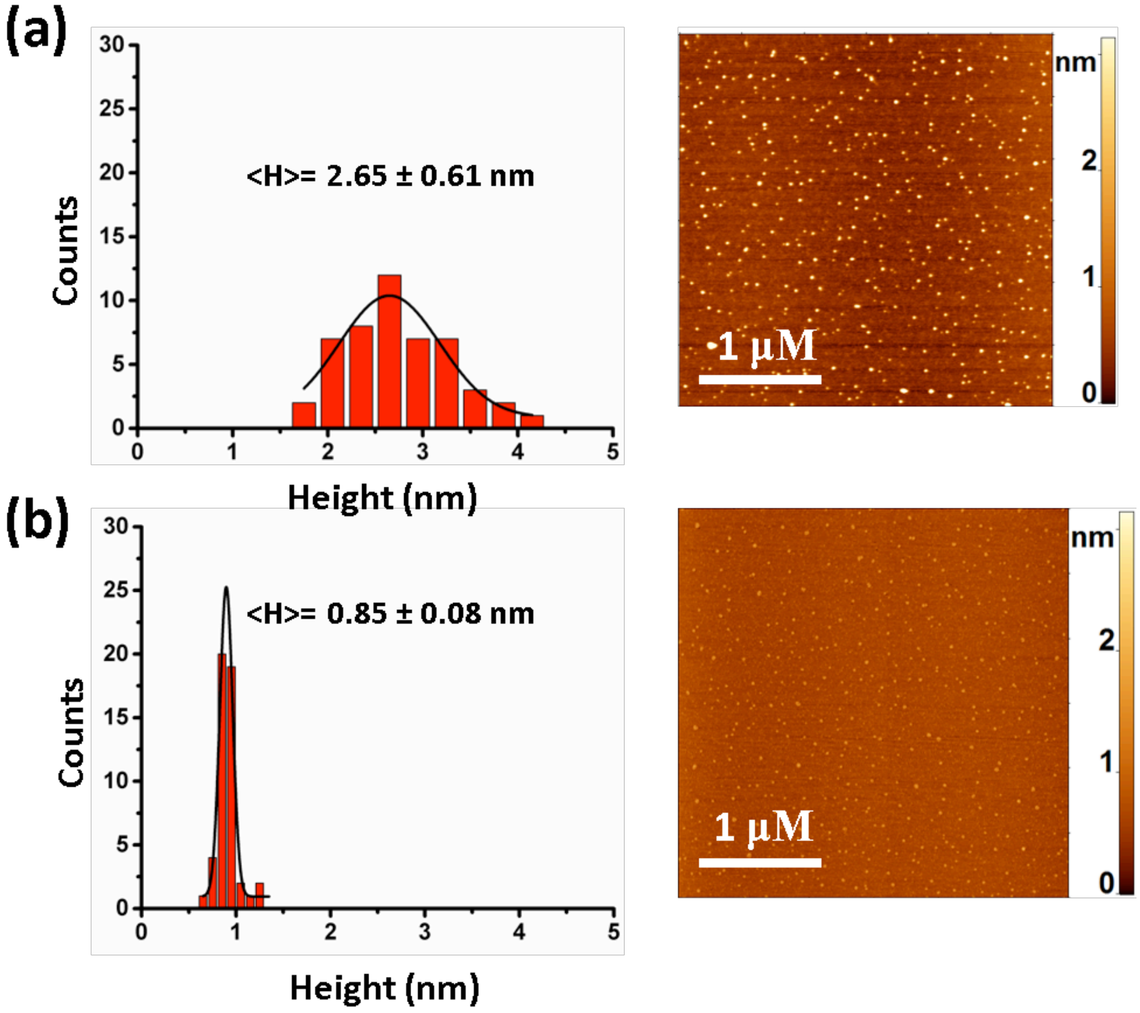

Figure S1. Height analysis for the aggregates from (a) hairpin only and (b) mixture of hairpin:monomer (1:1) upon 3 days of incubation at room temperature; (Left side) height histograms and (right side) topographic images. 50 particles from each set experiments were analyzed for height statistics. The histograms were fitted with Gaussian function and values are presented as mean ± S.D.

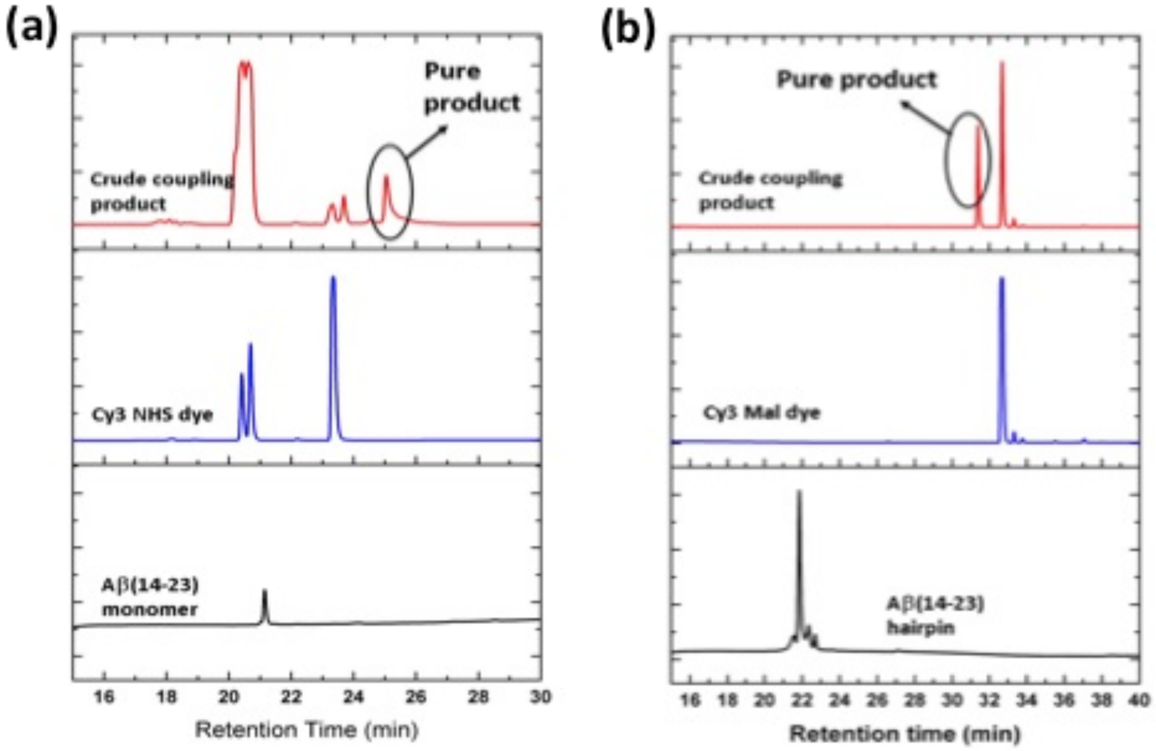

Figure S2. HPLC data representing isolation of Cy3 labeled monomer (a), and hairpin (b). The black lines, blue lines and red lines indicate the monomer or hairpin only, Cy3 NHS or Cy3 Mal and crude product of Cy3 conjugated monomer or hairpin respectively. The peak showing by black circle corresponds to Cy3 conjugated monomer or hairpin.

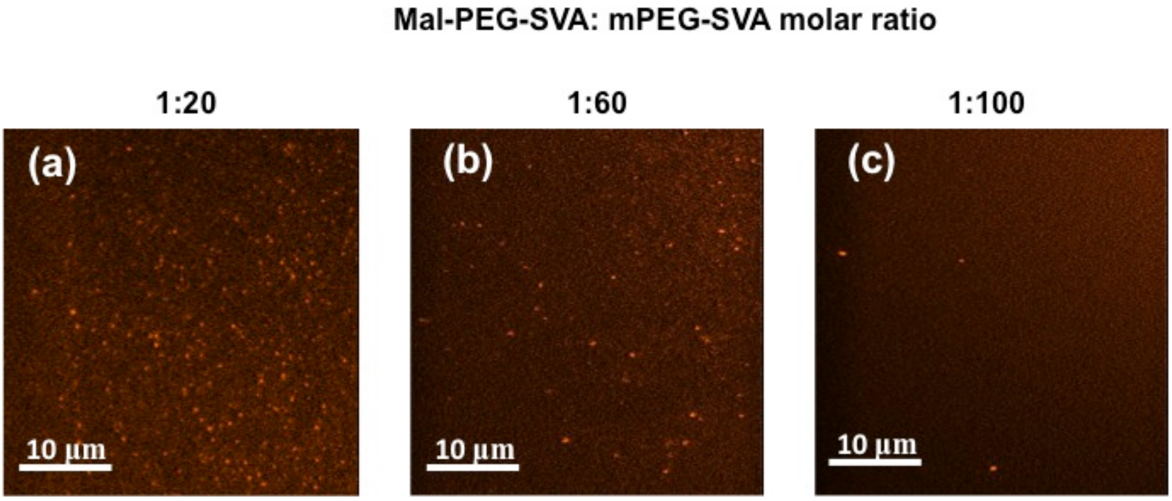

Figure S3. Optimization of Mal-PEG-SVA and mPEG-SVA molar ratio in the TAPIN experiment. The surfaces were treated with different ratio of Mal-PEG-SVA and mPEG SVA, followed by covalently immobilization of Cy3 labeled monomer. (a), (b) and (c) show TIRF images of the surfaces corresponds to PEG ratio 1:20, 1:60 and 1:100 respectively.

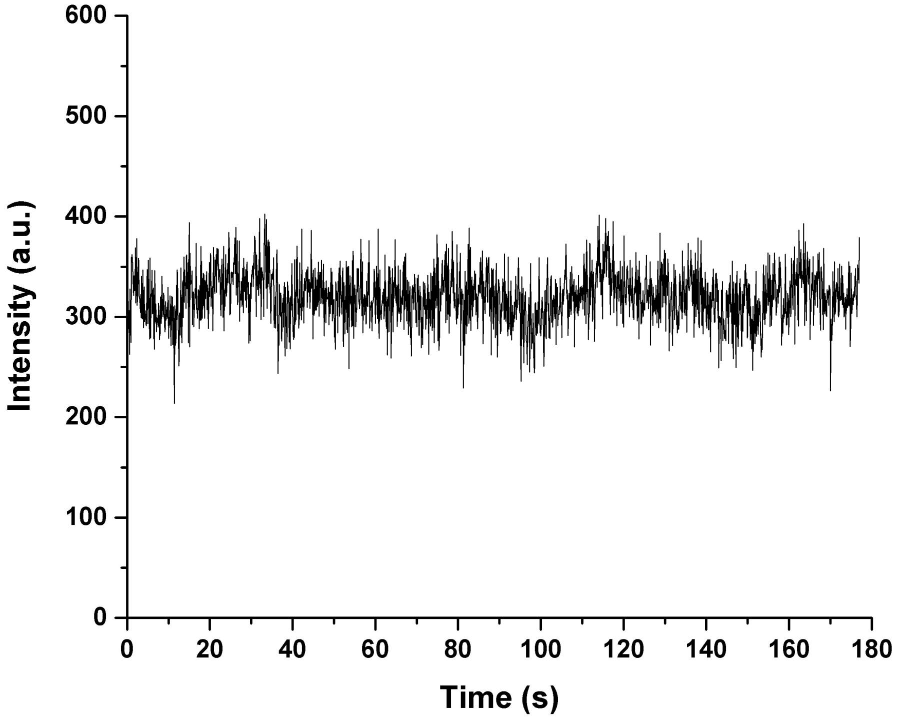

Figure S4. A typical time trajectory (after background correction) from the surface attached (covalently) Cy3 labeled monomer, showing no photo-bleaching and blinking in fluorescence time trajectory up to 3 min.

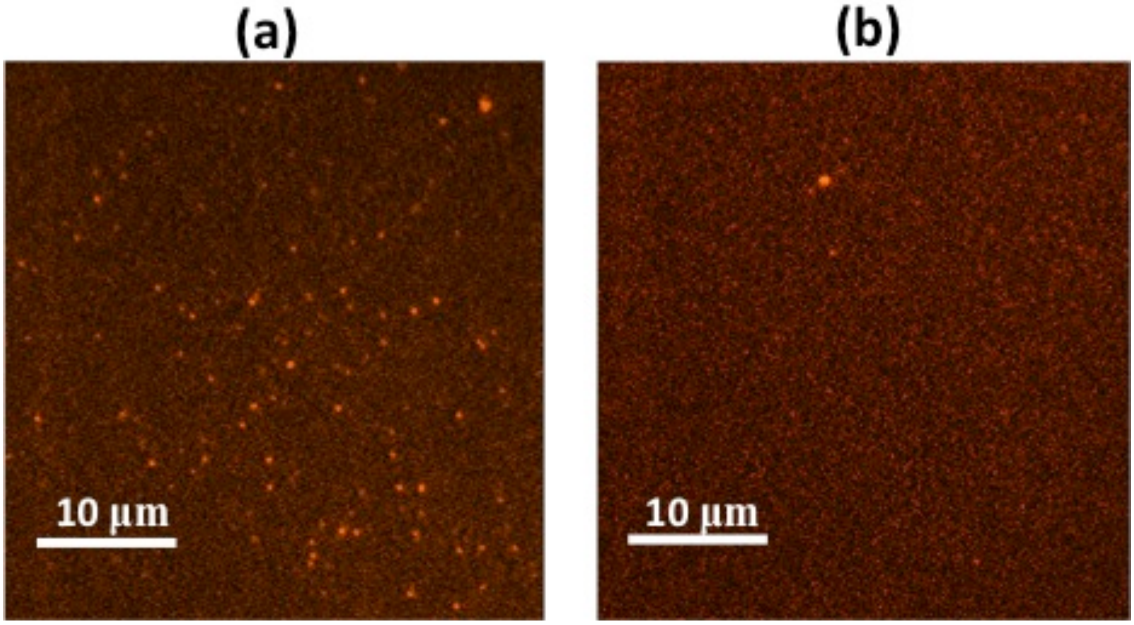

Figure S5. Comparison of specific vs. non-specific interactions; (a) Specific interaction of Cy3 labeled hairpin with the surface containing Aβ(14-23) monomers, showing a considerable number of hairpin-monomer complexes, (b) non-specific interaction with mPEG surface showing very few bright spots.

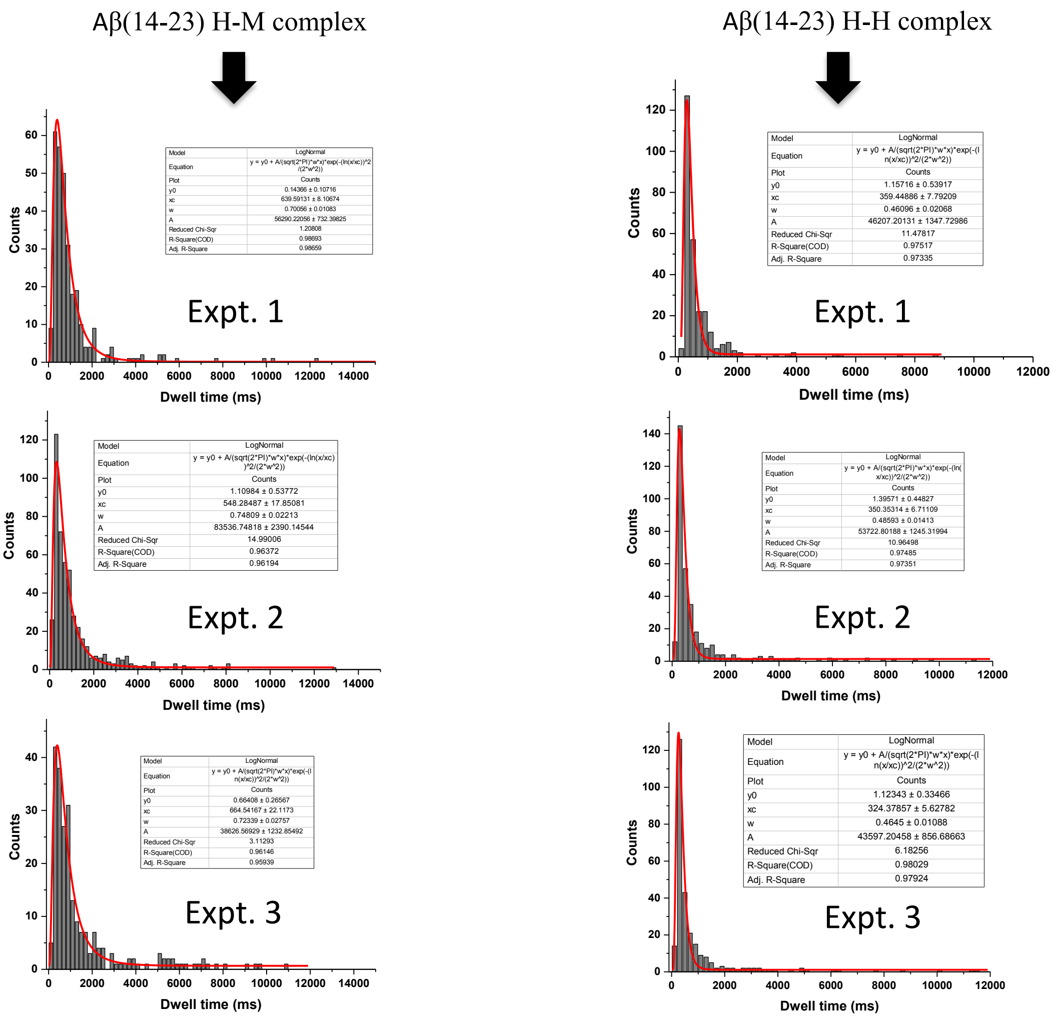

Figure S6. Lifetime histograms for the H-M (left column) and H-H complexes (right column) obtained from three independent experiments. Red lines indicate Lognormal fitting.

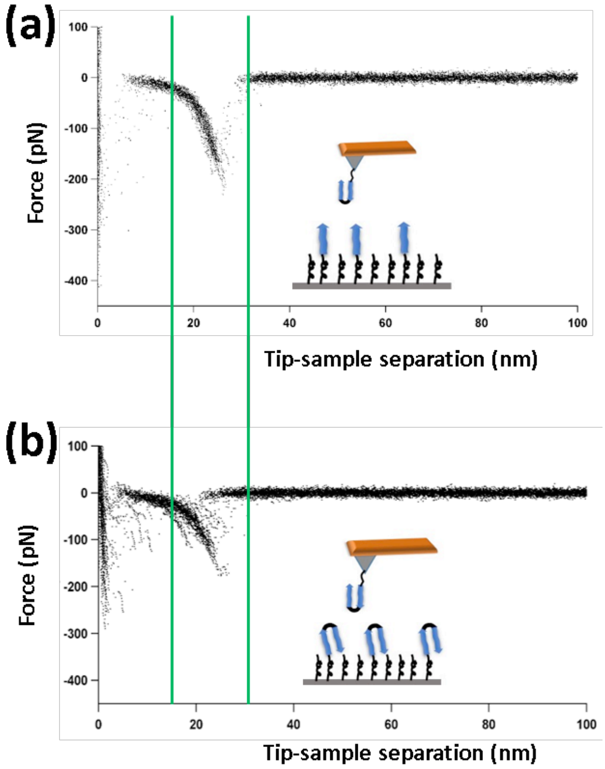

Figure S7. Overlay of force-distance curves obtained from AFM force spectroscopy for (a) H-M and (b) H-M complexes. The maximum force peaks are at a confined region shown by two vertical lines. The insets show experimental set up.

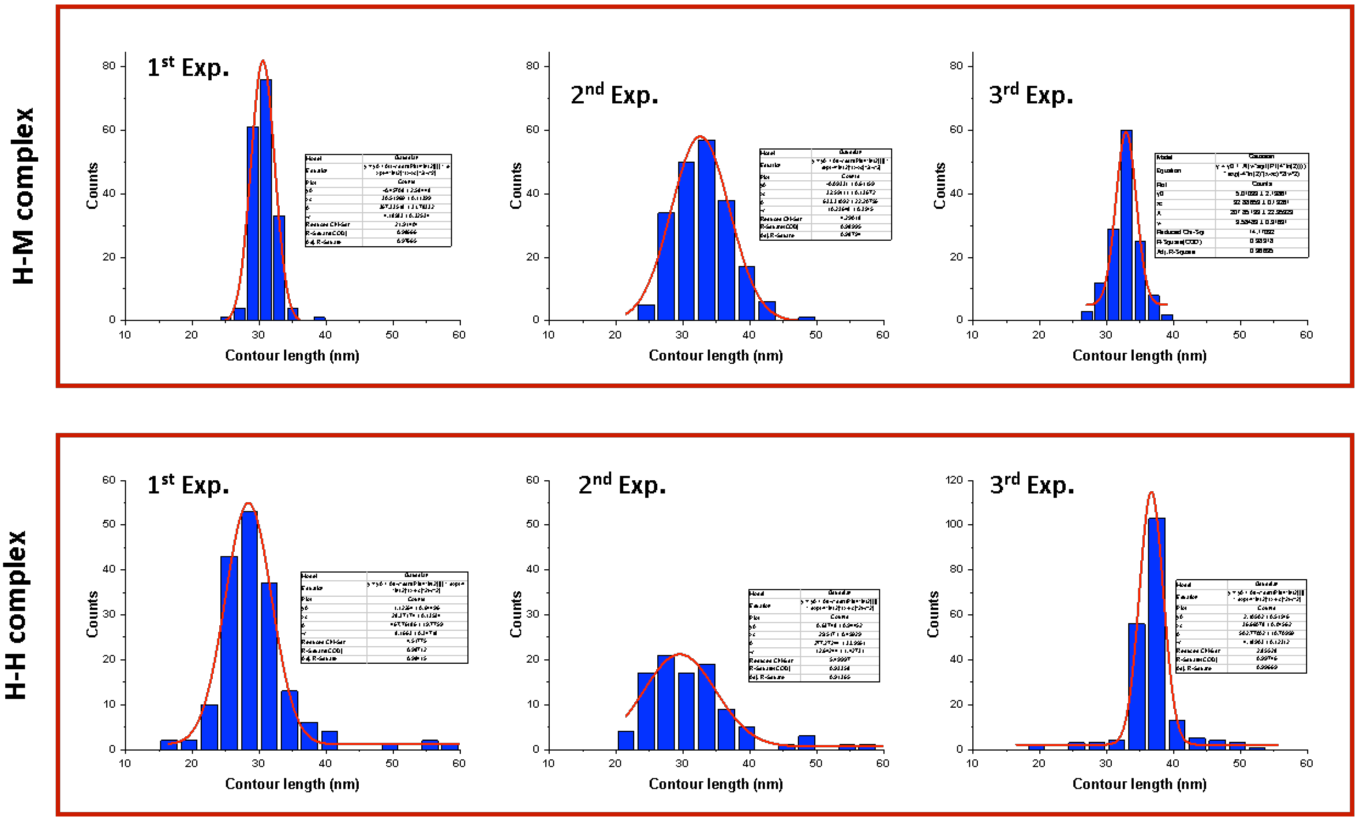

Figure S8. Contour length distributions from three independent experiments; H-M complexes (upper panel) and H-H complexes (lower panel). Red lines indicate Gaussian fitting.

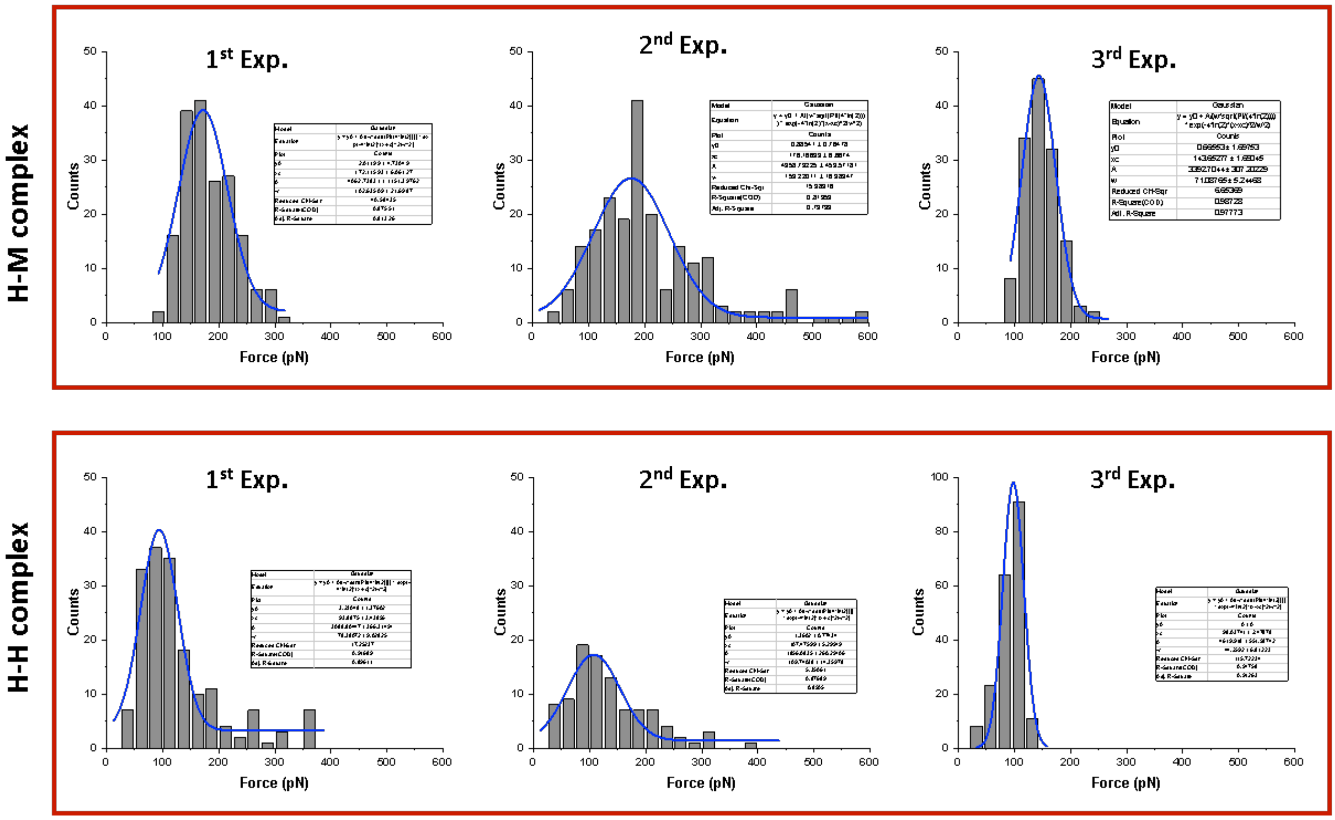

Figure S9. Force distributions for the H-M (upper panel) and H-H complexes (lower panel), obtained from three independent experiments. Blue lines indicate Gaussian fitting.

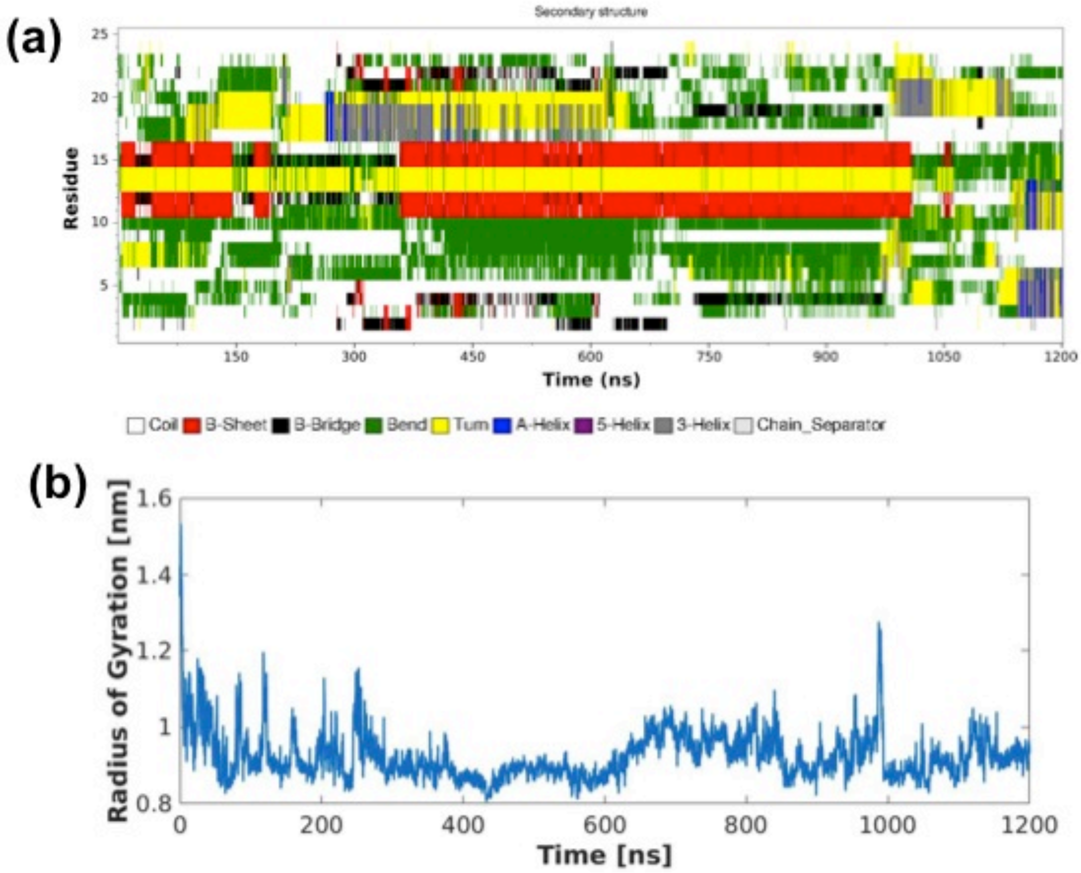

Figure S10. MD simulation of hairpin structure; (a) DSSP graph illustrating dynamics of secondary structure over time. The YNGK loop induces stable β−sheet structure at E10-D11-K12 and G14-K15-H16. (b) Change in the radius of gyration with respect to time.

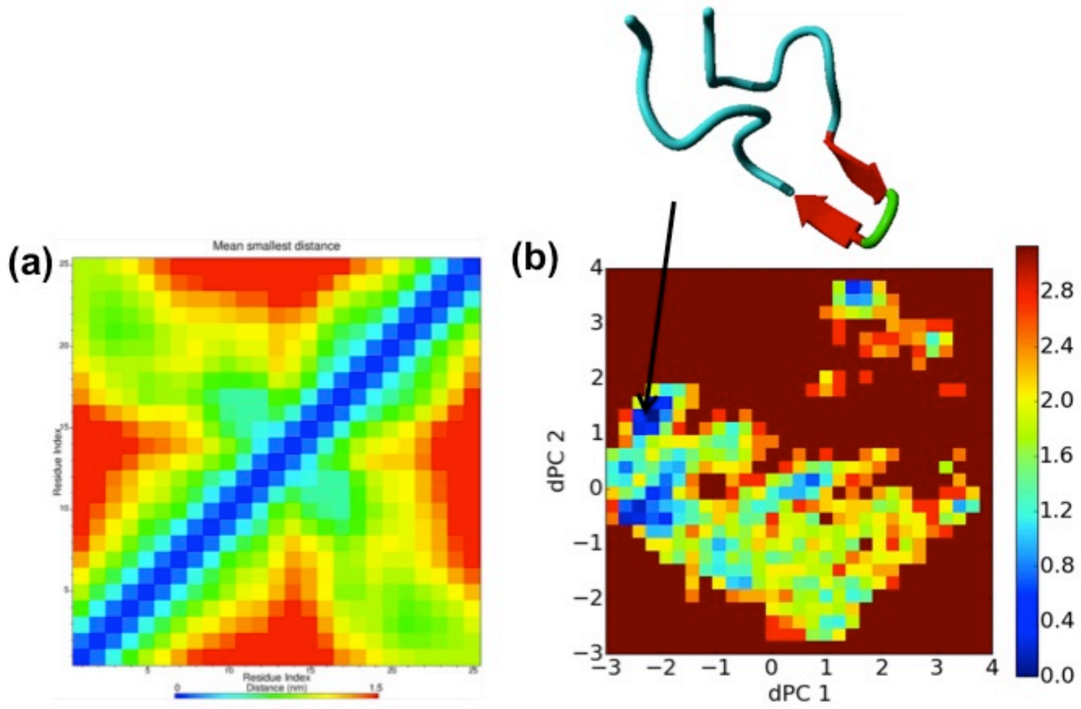

Figure S11. MD simulation of the hairpin structure. (a) Peptide backbone interaction map; the cross-diagonal interaction pattern clearly indicates a collapsed hairpin conformation. (b) Free energy landscape for the 1.2 μs MD simulation of hairpin peptide. The structure for the lowest energy minimum is shown in cartoon representation. Color bar is in units of k_BT.

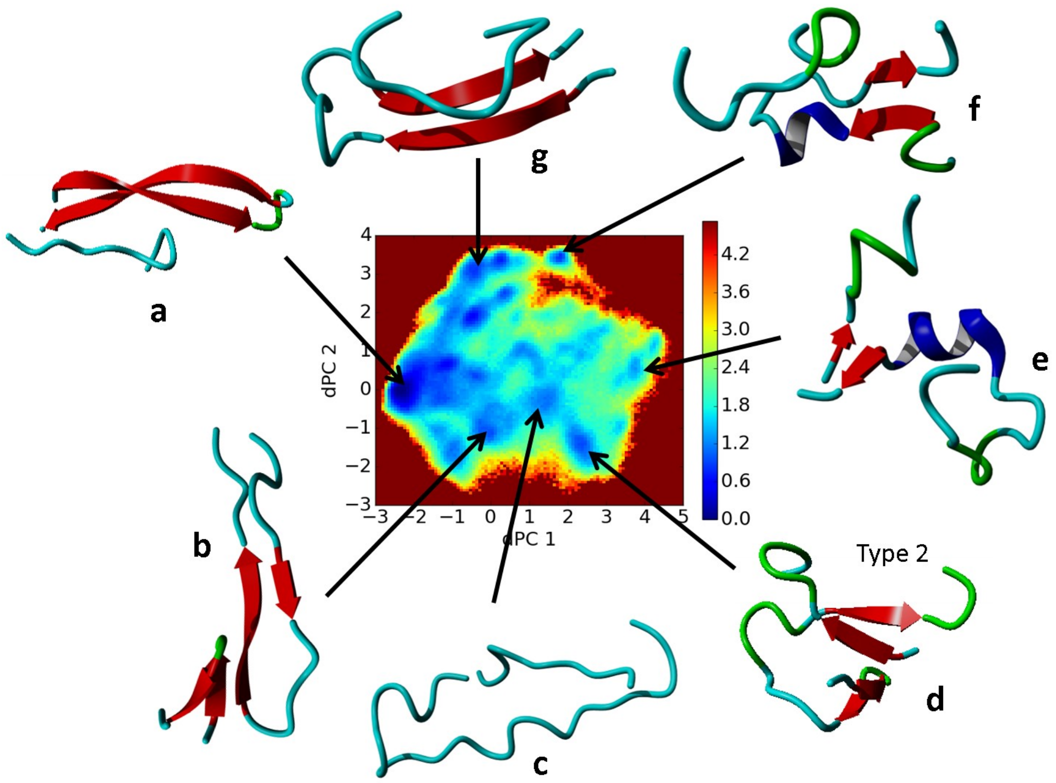

Figure S12. Energy landscape according to dihedral principal component analysis for 500 ns aMD simulation for the H-M complex. Selected minima are highlighted with the arrows and the most representative structures are shown for the respective minima (a-g). Color bar is in units of k_BT.

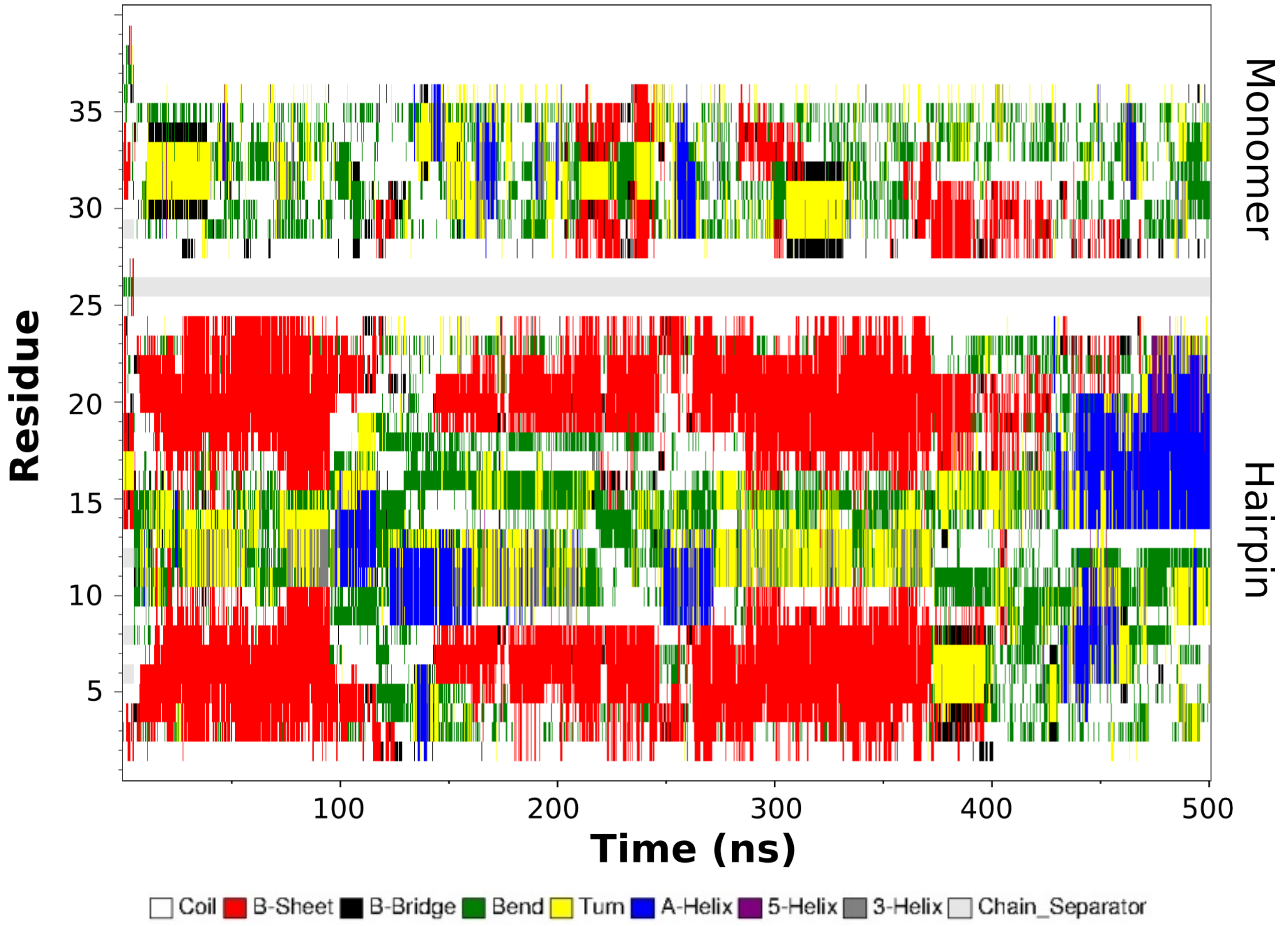

Figure S13. DSSP analysis shows the change in secondary structures of monomer and hairpin during aMD simulation for the H-M complex (structure in Fig. S12).

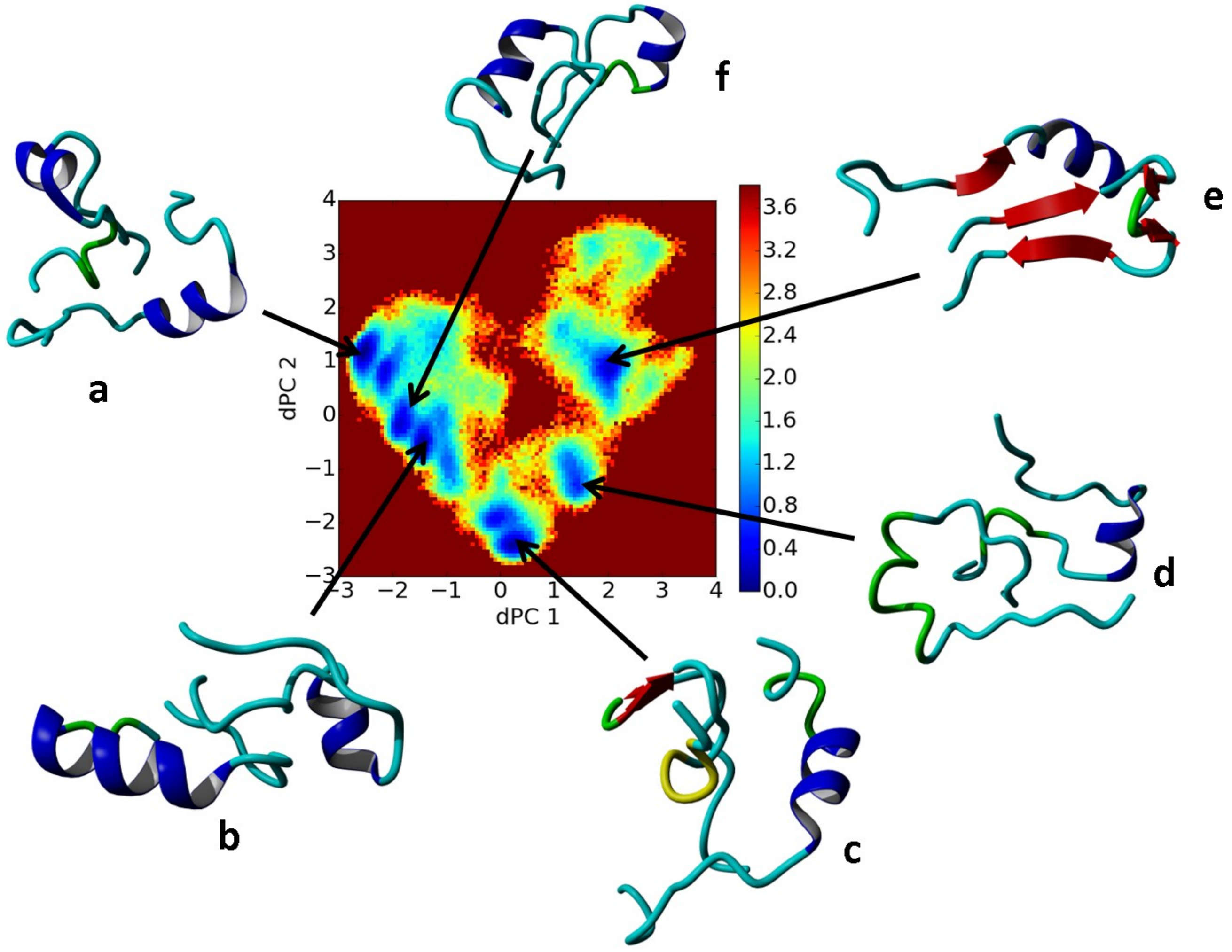

Figure S14. Free energy landscape, according to dPC analysis, after 500 ns aMD simulation for the H-H complex. The most representative structures are shown for some of the minima (a-f). Color bar is in units of k_BT.

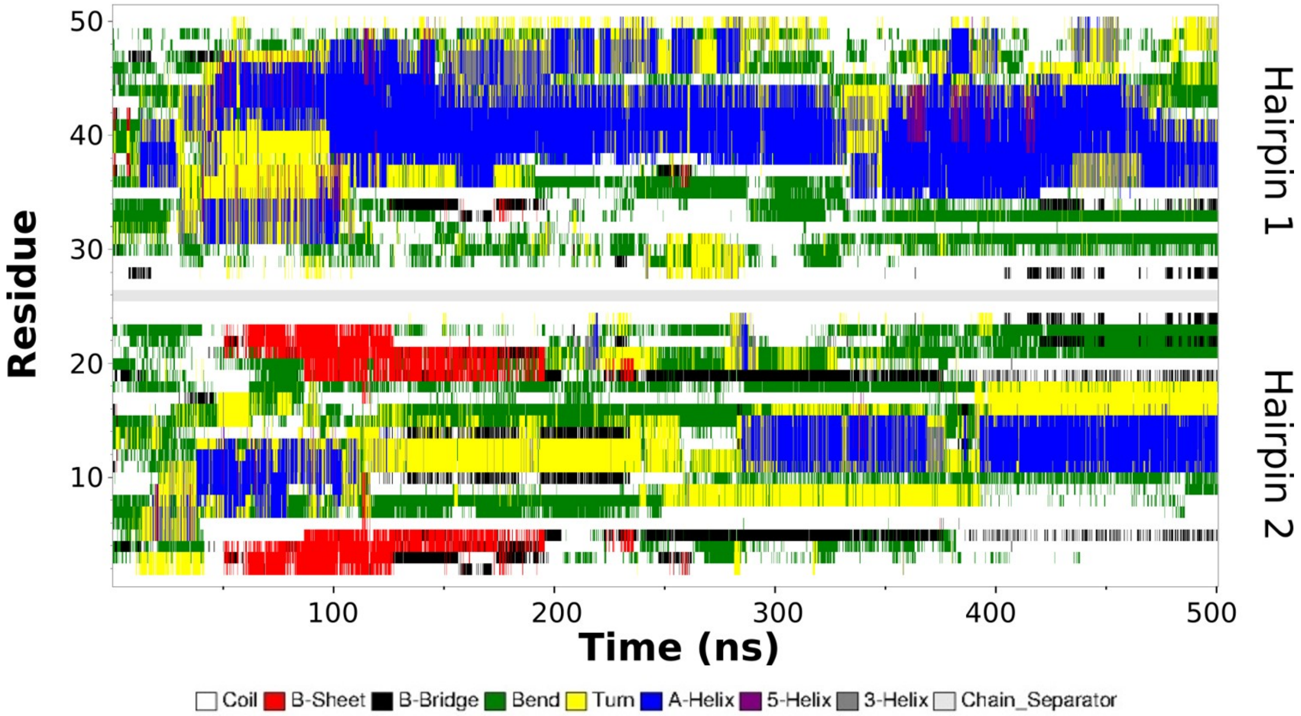

Figure S15. DSSP analysis shows the change in secondary structures of individual hairpin during aMD simulation for the H-H complex (structure in Fig. S14).

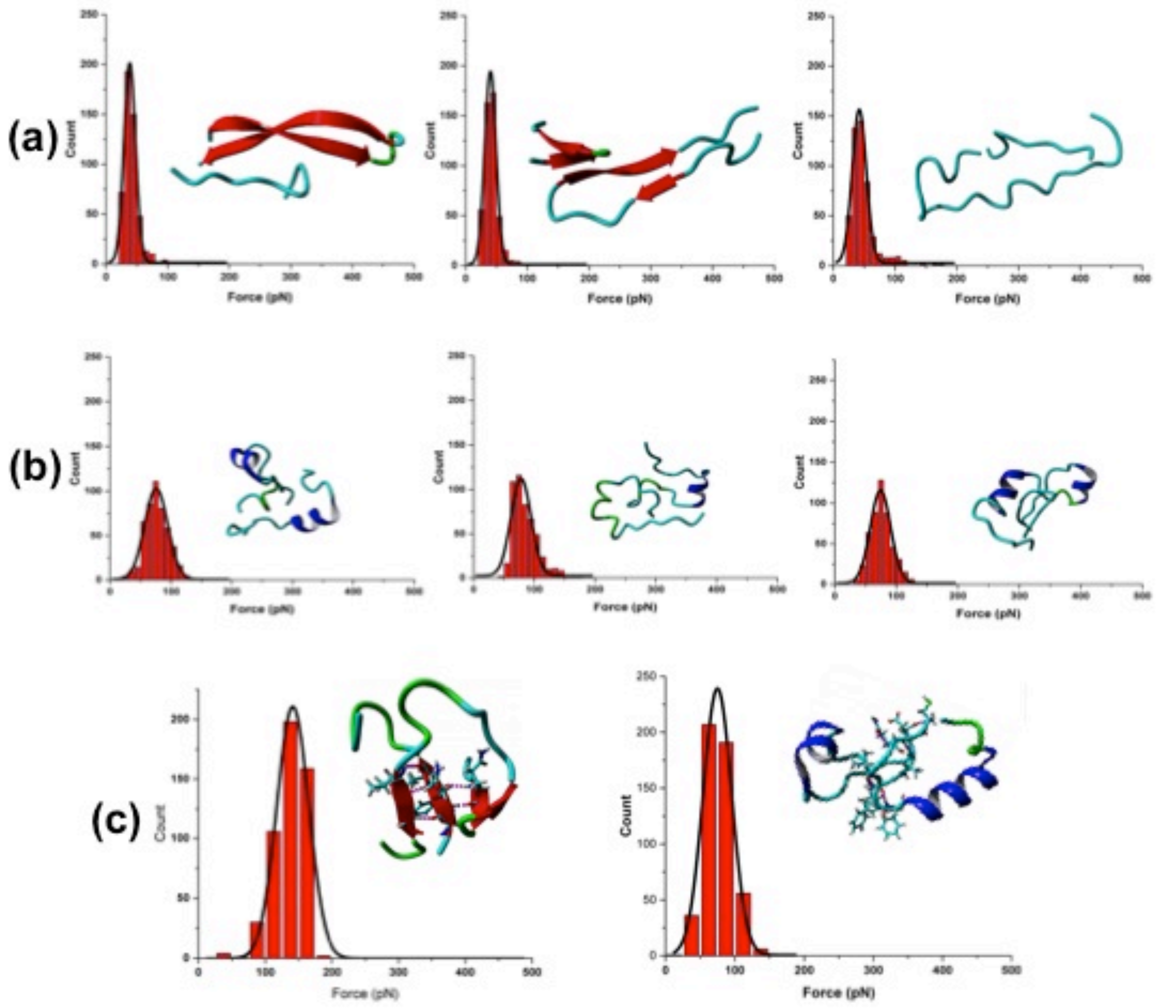

Figure S16. MCP force histograms for the additional structures of the H-M and H-H complexes. (a) MCP force histograms for three structures of H-M complexes and corresponding structures are shown in the insets. (b) MCP force histograms for three structures of H-H complexes and corresponding structures are shown in the insets. (c) Histograms and predicted structure for H-M and H-H complexes. The black curves indicate Gaussian fitting.

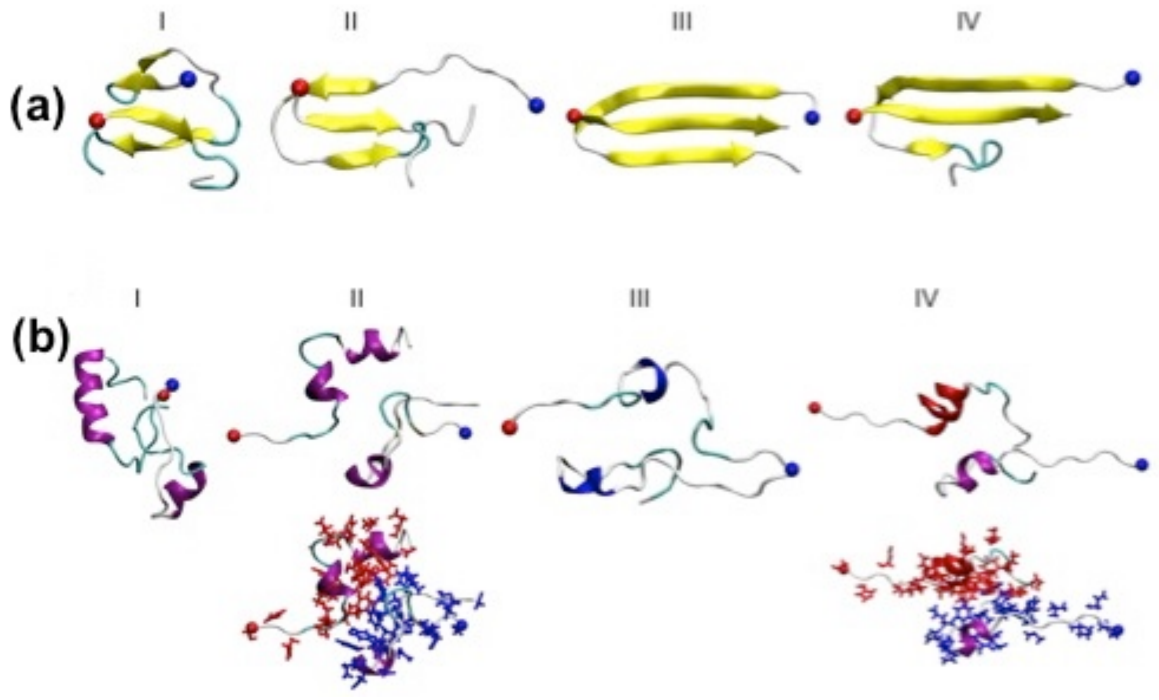

Figure S17. Rupture processes for H-M and H-H complexes during MCP experiments. (a) The rupture process for H-M complex producing 140 pN force. The hairpin transitions to a horseshoe shape, which allows the monomer to interact with a larger number of residues leading to the formation of an extended β sheet. Dissociation occurs when the sheet is destabilized. (b) Rupture of the H-H complex as the MCP experiment progresses, the force is 90 pN. Force induces conformational change in the complex leading to the loss of secondary structure. As the experiment progresses the complex is stabilized by extensive interactions of the side chains. Rupture finally occurs when the side chain interactions are disrupted. Blue and red spheres represent the pulling groups.

Table S1. Lifetime values obtained from three independent single-molecule fluorescence experiments for the H-M and H-H complexes.

		Measured lifetime mean from Lognormal fitting (ms)	Average lifetime (mean ± S.D) from three experiments (ms)
**H-M complex**	Expt. 1	639	664 ± 61
	Expt. 2	548	
	Expt. 3	664	
**H-H complex**	Expt. 1	359	344 ± 18
	Expt. 2	350	
	Expt. 3	324	

Table S2. AFM force spectroscopy data obtained from three independent experiments for both the H-M and H-H complexes.

		Rupture force, mean from Gaussian fitting (pN)	Average rupture force (mean ± standard deviation) from three experiments (pN)
**H-M complex**	Expt. 1	176	164 ± 17
	Expt. 2	172	
	Expt. 3	144	
**H-H complex**	Expt. 1	107	100 ± 6
	Expt. 2	94	
	Expt. 3	98	

Supporting movies

1. **Movie S1: Assembly-disassembly TIRF video for H-M complexes**
2. **Movie S2. Assembly-disassembly TIRF video for H-H complexes**
3. **Movie S3. Photo bleaching test**

References

1 Lovas, S., Zhang, Y., Yu, J. & Lyubchenko, Y. L. Molecular Mechanism of Misfolding and Aggregation of A β (13‑23). *The Journal of Physical Chemistry B* **117**, 6175-6186, doi:10.1021/jp402938p (2013).

2 Pierce, L. C., Salomon-Ferrer, R., Augusto, F. d. O. C., McCammon, J. A. & Walker, R. C. Routine Access to Millisecond Time Scale Events with Accelerated Molecular Dynamics. *Journal of chemical theory and computation* **8**, 2997-3002, doi:10.1021/ct300284c (2012).

3 Zhang, Y., Hashemi, M., Lv, Z. & Lyubchenko, Y. L. Self-assembly of the full-length amyloid A[small beta]42 protein in dimers. *Nanoscale* **8**, 18928-18937, doi:10.1039/C6NR06850B (2016).

4 Mu, Y., Nguyen, P. H. & Stock, G. Energy landscape of a small peptide revealed by dihedral angle principal component analysis. *Proteins* **58**, 45-52, doi:10.1002/prot.20310 (2005).

5 Zhang, Y. & Lyubchenko, Yuri L. The Structure of Misfolded Amyloidogenic Dimers: Computational Analysis of Force Spectroscopy Data. *Biophysical journal* **107**, 2903-2910, doi:10.1016/j.bpj.2014.10.053.
